# A Polyhydroxybutyrate-Supported Xerogel Biosensor for Rapid BOD Mapping and Integration with Satellite Data for Regional Water Quality Assessment

**DOI:** 10.3390/gels11110849

**Published:** 2025-10-24

**Authors:** George Gurkin, Alexey Efremov, Irina Koryakina, Roman Perchikov, Anna Kharkova, Anastasia Medvedeva, Bruno Fabiano, Andrea Pietro Reverberi, Vyacheslav Arlyapov

**Affiliations:** 1 BioChemTech Research Center, Tula State University, Pr. Lenina 92, 300012 Tula, Russiaefremovalexeychem@yandex.ru (A.E.); irina_koryakinaa@mail.ru (I.K.);; 2 Department of Civil, Chemical and Environmental Engineering (DICCA), Genoa University, Via Opera Pia, 15, 16145 Genoa, Italy; 3 Department of Chemistry and Industrial Chemistry (DCCI), Genoa University, Via Dodecaneso, 31, 16146 Genoa, Italy

**Keywords:** sol–gel, xerogel, biosensor, BOD, environmental monitoring, polyhydroxybutyrate

## Abstract

The growing threat of organic pollution to surface waters necessitates the development of rapid and scalable monitoring tools that transcend the limitations of the standard 5-day biochemical oxygen demand (BOD_5_) test. This study presents a novel approach by developing a highly stable and rapid BOD biosensor based on the microorganism *Paracoccus yeei*, immobilized within a sol–gel-derived xerogel matrix synthesized on a polyhydroxybutyrate (PHB) substrate. The PHB-supported xerogel significantly enhanced microbial viability and sensor stability. This biosensor demonstrated a correlation (R^2^ = 0.93) with the standard BOD_5_ method across 53 diverse water samples from the Tula region, Russia, providing precise results in just 5 min. The second pillar of our methodology involved analyzing multi-year Landsat satellite imagery via the Global Surface Water Explorer to map hydrological changes and identify zones of potential anthropogenic impact. The synergy of rapid ground-truth biosensor measurements and remote sensing analysis enabled a comprehensive spatial assessment of water quality, successfully identifying and ranking pollution sources, with wastewater discharges and agro-industrial facilities constituting the most significant factors. This work underscores the high potential of PHB–xerogel composites as efficient immobilization matrices and establishes a powerful, scalable framework for regional environmental monitoring by integrating advanced biosensor technology with satellite observation.

## 1. Introduction

The problem of water pollution by organic waste is one of the most pressing environmental issues worldwide [1,2]. According to recent assessments, climate change and human activities increasingly threaten lakes, which store 87% of the Earth’s surface freshwater [3,4]. With growing population and urbanization, the level of water pollution will only increase. In particular, small rivers and reservoirs are subject to significant anthropogenic impact, leading to eutrophication and the deterioration of ecosystems [5,6]. Addressing this problem requires the implementation of new purification and waste management technologies, as well as improvements to existing water quality monitoring systems [7,8]. Biochemical oxygen demand (BOD) is one of the most widely used parameters for assessing the purity of aqueous environments and is defined as the amount of oxygen required for the biochemical oxidation of organic substances contained in a sample [9]. The conventional method for BOD determination requires incubating an oxygen-saturated sample for 5, 10, or 20 days (BOD_5_, BOD_10_, or BOD_20_, respectively). This standard method, which provides results with a significant delay, is incapable of facilitating real-time environmental monitoring today [10]. Although the BOD_5_ assay is one of the most common tests in water analysis, the variance of results in certified laboratories reaches 20% (for intra-laboratory control), and this value increases in inter-laboratory comparisons. This is associated with the instability of the microbial consortia used in the tests [11]. These limitations stimulate the search for rapid alternatives, among which biosensor technologies are the most promising [12,13].

**Figure 1:**
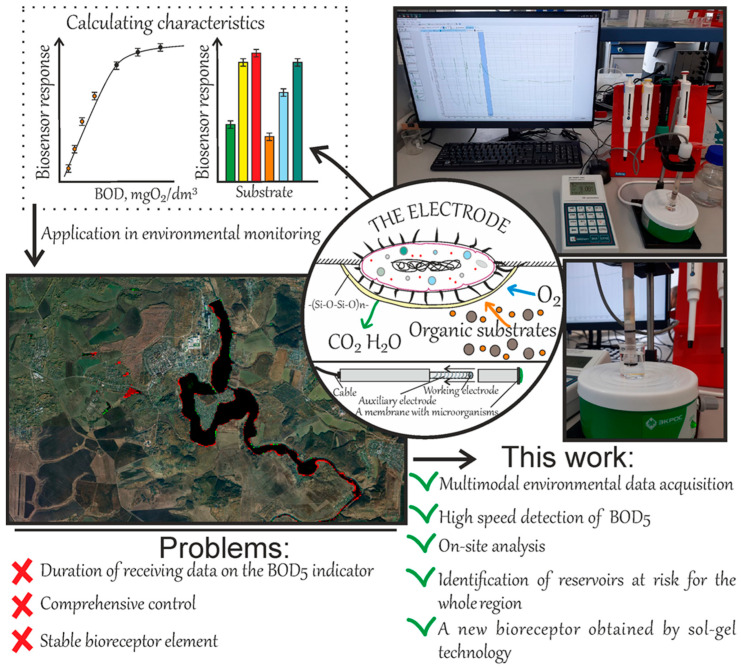
Principle of operation and advantages of the “Expert-009” biosensor for identifying regional environmental hazards. The system integrates a xerogel-based bioreceptor, fabricated via sol–gel technology, with long-term satellite data analysis for comprehensive monitoring.

An alternative to the standard method is the rapid determination of BOD using biosensor analyzers based on microorganisms capable of oxidizing a wide spectrum of organic compounds [14,15,16]. The fundamental difference in this method from the standard one is the significant reduction in analysis time from 5 days to 10–20 min [17]. The most commonly used primary transducer in such analyzers is an amperometric sensor with a gas-permeable membrane (Clark-type electrode), as it provides rapid, real-time information, high reliability, and low cost [18]. Furthermore, a BOD analyzer must provide automatic temperature compensation for readings, as oxygen solubility and membrane permeability are temperature-dependent [19]. The principle of BOD measurement using biosensors (Figure 1) is based on the oxidation of an introduced substrate by microorganisms in the measuring cell. This leads to an increase in the respiratory activity of the cells, causing a decrease in oxygen concentration near the electrode, which is subsequently detected by the oxygen electrode [20]. The correlation coefficient between BOD values determined using the biosensor and the standard method ranges from 0.6 to 0.99. However, the large number of publications regularly emerging on this topic indicates that characteristics that would halt the process of further investigation have not yet been achieved. Current challenges in BOD sensor development include improving analysis sensitivity, increasing the lifetime of biological material in the biosensor’s receptor elements, and simplifying analyzer maintenance requirements. These challenges can be addressed through the application of xerogels for biomaterial immobilization, obtained via sol–gel technology on various substrates. Such matrices are of significant interest to the scientific community due to their ability to form a stable, protective, and biocompatible shell around the biological material. They are gels dried at room temperature, which are attractive owing to their mechanical stability, large surface area, ease of production, and biocompatibility [21]. Their potential has been noted in medicine as encapsulating materials [22] and antibacterial coatings [23], in tissue engineering [24,25], as well as for the immobilization of enzymes, antibodies, and cells for biosensors [26,27,28]. The most common approach for obtaining xerogels is sol–gel technology [29]. This method is convenient because synthesis is conducted at room temperature on various surfaces, and the porosity and characteristics of the final material can be tuned by altering the synthesis conditions and initial precursors [30]. To facilitate ease of use and enhance cell attachment, various substrates can be employed, for example, using biopolymers. Thus, polyhydroxyalkanoates, particularly poly(3-hydroxybutyrate) (PHB), can be excellent candidates for this purpose, as they are extremely biocompatible and easy to process into films of various shapes, thicknesses, and porosity [31].

Despite the high accuracy of data obtained in the laboratory or via in situ measurements, satellite imagery also represents a valuable source of information. It serves as a crucial tool for modern environmental monitoring, providing a comprehensive assessment of water bodies at global and regional levels [32,33,34]. Its key advantage over traditional methods lies in its capacity for continuous observation of the dynamics of aquatic ecosystems, including processes of pollution, eutrophication, and anthropogenic impact, with high temporal frequency [35,36]. A significant breakthrough in this area was achieved in the work by Pekel et al. [37], where the authors developed an innovative methodology for analyzing surface waters based on a 32-year archive of Landsat (5, 7, 8) satellite imagery. The application of expert classification algorithms allowed for achieving 99% accuracy in detecting water bodies as small as 30 × 30 m, including the analysis of seasonal fluctuations and long-term changes. The researchers’ created platform, the Global Surface Water Explorer, demonstrated its effectiveness in identifying large-scale hydrological changes, such as the disappearance of 90,000 km^2^ of permanent water bodies and the formation of 184,000 km^2^ of new ones between 1984 and 2015. The method’s capabilities for analyzing regional water systems, including the small and large rivers of the Tula region, are of particular scientific and practical value. Despite their modest size, small watercourses play a critically important role in forming the hydrological regime of large river systems, acting as natural filters and regulators of water balance [38]. However, they are also the most vulnerable to anthropogenic impact and require special attention in monitoring.

Earth Observation (EO) from satellites has the potential to provide comprehensive, rapid and inexpensive information about water bodies, integrating in situ measurements [39]. An advanced direction in the development of environmental control methods is the integration of satellite monitoring with biosensor technologies [40,41]. This combination merges the global coverage of remote sensing with the high accuracy of in situ measurements, creating a reliable foundation for water resource management. Despite existing limitations related to the spatial resolution of imagery and the influence of cloud cover, the proposed approach opens new possibilities for the rapid identification of environmentally critical situations [42,43,44]. In this context, the aim of the present study was to develop a biosensor system based on a xerogel matrix produced by sol–gel technology with immobilized microorganisms of *Paracoccus yeei* VKM B-3302 (hereinafter referred to as *P. yeei*). The developed system was tested on real water bodies in the Tula region. The effectiveness of the combined use of the biosensor analyzer and satellite data for addressing environmental monitoring tasks and identifying environmentally critical areas across the entire region was evaluated.

## 2. Results and Discussion

Conventional BOD monitoring methods, which take up to five days, fundamentally limit the possibilities for rapid water quality control. In this work, we present an innovative biosensor approach, the key element of which is a xerogel matrix produced by sol–gel technology. This matrix serves for the effective immobilization of microorganisms, providing them with a stable and biocompatible environment, which significantly increases the biosensor’s lifetime and sensitivity. The combination of this rapid method, which reduces the analysis time to 5 min, with the analysis of multi-year satellite data, has enabled the implementation of a comprehensive approach for identifying and ranking environmental threats of organic pollution in surface waters at a regional level. This synergistic method not only demonstrates a correlation (R^2^ = 0.93) during validation on 53 samples but also vividly illustrates the potential of gel-based materials for creating effective environmental monitoring tools.

### 2.1. Fabrication and Characterization of the Bioreceptor Element

**Figure 2:**
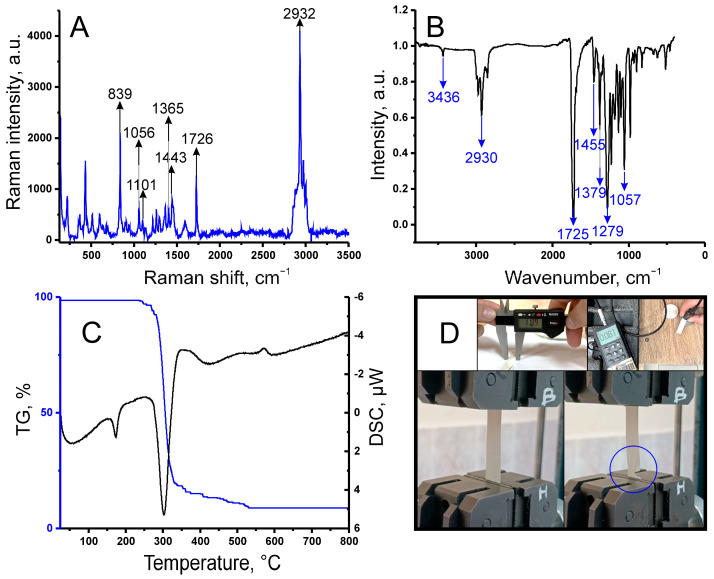
Physicochemical characterization of the PHB film for the bioreceptor element. (**A**) Raman spectrum of the PHB film. (**B**) FT-IR spectrum of the PHB film. (**C**) TG and DSC curves of the PHB sample. (**D**) Physicomechanical testing of the PHB film.

The first stage in creating the biosensor involved fabricating a biocompatible and mechanically stable substrate. For this purpose, the strain *Cupriavidus necator* VKM B-3386 was used, cultivated under nitrogen-limiting conditions to ensure efficient synthesis of intracellular poly(3-hydroxybutyrate) (PHB). Subsequent extraction of the polymer with chloroform and precipitation with hexane yielded purified PHB, which was then used to produce films via the solution casting method. The structure and purity of the obtained PHB were confirmed by a suite of physicochemical methods (Figure 2).

**Figure 3:**
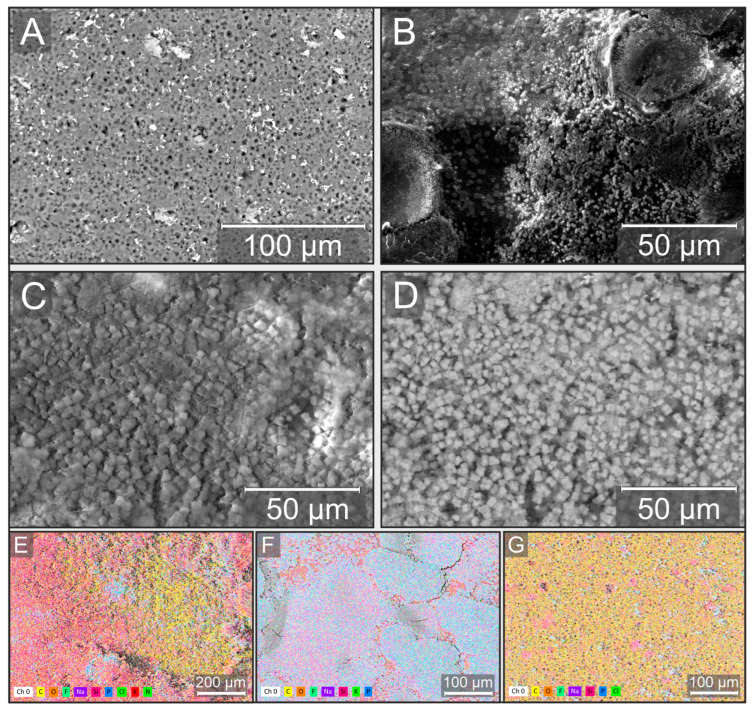
Morphology and elemental composition of the bioreceptor element. (**A**) Pristine PHB film. (**B**–**D**) PHB film with the immobilized *P. yeei*/xerogel composite in secondary (**B**,**C**) and backscattered (**D**) electron modes. (**E**–**G**) Energy-dispersive X-ray (EDX) spectroscopy analysis of the composite (**E**), pure xerogel with cells (**F**), and pristine PHB film (**G**). Scale bars: (**A**) 100 μm, (**B**–**D**) 50 μm, (**E**) 200 μm, (**F**,**G**) 100 μm.

Raman spectroscopy (Figure 2A) identified characteristic bands at 1726 cm^−1^ (C=O) and 1101 cm^−1^ (C–O–C), which aligned with the absorption bands observed by Fourier-transform infrared (FT-IR) spectroscopy (Figure 2B), such as the C=O stretch at 1725 cm^−1^. Definitive proof of the polymer’s molecular structure was provided by ^1^H and ^13^C nuclear magnetic resonance (NMR) spectra. Detailed results of the spectroscopic analyses can be found in the Supplementary Materials. The thermal behavior of the PHB films was investigated by thermogravimetric analysis coupled with differential scanning calorimetry (TGA-DSC) (Figure 2C). The observed endothermic melting peak at 172.9 °C and the onset of thermal decomposition at ~240 °C indicate sufficient thermal stability of the material, which is further supported by the significant interval (~70 °C) between these processes. The mechanical properties of the films (Figure 2D) (tensile strength of 6.5 ± 0.5 MPa, Young’s modulus of 250 ± 10 MPa) were found to be typical for non-oriented PHB and confirmed its suitability for use as a structural substrate material in the biosensor. Graphs and descriptions of the physico-mechanical analyses of the PHB film are provided in the Supplementary Materials. To confirm the successful formation of the bioreceptor element and to study the distribution of microorganisms within the xerogel matrix, an analysis was performed using scanning electron microscopy (SEM) and energy-dispersive X-ray spectroscopy (EDX) (Figure 3).

SEM analysis showed that the surface of the pure PHB substrate has a distinct porous structure (Figure 3A), which, as expected, results from solvent evaporation during the casting process. This porosity provides a high specific surface area and promotes effective adhesion of the xerogel coating. Images of the modified substrate (Figure 3B) show that the xerogel forms a continuous layer on the PHB surface. A key result was the visualization of the immobilized *P. yeei* cells. In the backscattered electron (BSE) imaging mode (Figure 3D), microbial cells are clearly observed, whereas they are indistinguishable in the secondary electron (SE) mode (Figure 3C). This provides direct evidence of their successful encapsulation within the volume of the xerogel matrix, rather than mere adsorption on the surface. The observed predominant accumulation of cells in the lower part of the xerogel layer (near the interface with the substrate) indicates their sedimentation during the gel formation process, resulting in a highly active biocatalytic layer in immediate proximity to the substrate surface. Elemental analysis by EDX (Figure 3E) confirmed the expected chemical composition of the system. The presence of silicon (Si) and oxygen (O) is attributed to the silicate matrix of the xerogel. The fluorine (F) and sodium (Na) peaks correspond to the use of sodium fluoride as a catalyst for the sol–gel process. The presence of phosphorus (P), potassium (K), and partially sodium (Na) is associated with the components of the phosphate-buffered solution used in preparing the cell suspension. Most importantly, the detection of nitrogen (N)—a key element of biomass—provides unambiguous confirmation of the presence of immobilized microorganisms within the coating. The dominance of carbon (C) in the spectrum is explained by its main source—the PHB substrate. This was further corroborated by control measurements of pure xerogel and the pure substrate (Figure 3F and Figure 3G, respectively). Thus, the comprehensive SEM-EDX analysis proved that the proposed fabrication method enables the creation of a stable bioreceptor element in which *P. yeei* cells are effectively encapsulated within a porous xerogel matrix firmly attached to the biopolymer substrate.

### 2.2. Evaluation of the Performance of the BOD Biosensor

**Figure 4:**
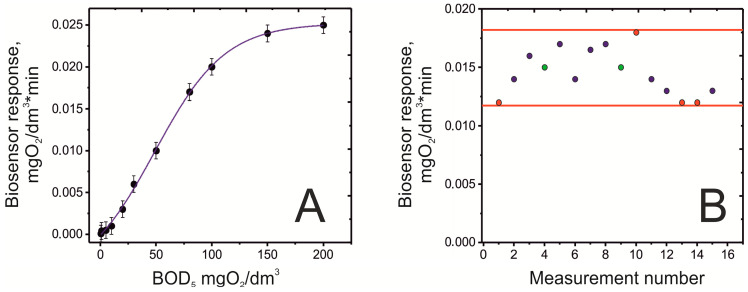
Calibration curve of the sensor response versus substrate concentration (**A**) and operational stability over 15 consecutive measurements of identical substrate concentration (**B**), reflecting the key characteristics of the biosensor based on *P. yeei* microorganisms immobilized in a xerogel matrix on a PHB substrate.

The key operational characteristics of the developed biosensor were determined for the finished bioreceptor element, which was based on the xerogel immobilization matrix. The main parameters of the biosensor were evaluated to determine its suitability for practical application. The calibration curve of the biosensor response (Figure 4A) (rate of oxygen consumption) versus BOD concentration was obtained using a standard glucose-glutamic acid (GGA) solution. It was established that the biosensor has a linear BOD determination range from 0.5 to 50 mg O_2_/dm^3^, which covers the typical values for most natural and polluted waters. The response time (time for a single measurement) was 5 min, which is three orders of magnitude shorter compared to the standard 5-day method. The bioreceptor element also features a broad substrate specificity, owing to the microorganisms isolated from activated sludge. This allows it to respond to most organic pollutant substrates that affect the BOD index and are present in water bodies. A critical parameter of reproducibility—the relative standard deviation (RSD)—did not exceed 15% for a series of measurements within the linear range, indicating acceptable stability of the biosensor element (Figure 4B). The low reagent consumption (measurement cell volume does not exceed 25 mL) and the lack of need for highly qualified personnel make the proposed methodology significantly more economical and efficient compared to the classical dilution method (Table 1).

Quantitative analysis of mass transfer limitations yielded an effectiveness factor (η) of 0.67, calculated from the ratio of oxygen consumption rates between immobilized and free cells. This value confirms moderate but acceptable diffusion constraints through the xerogel matrix, validating its suitability for biosensor applications. Details are provided in Supplementary Materials.

Thus, the use of sol–gel technology to create a xerogel matrix enabled the development of a biosensor element that not only provides high analytical performance (wide linear range, fast response time) but also possesses sufficient stability for practical application. The obtained characteristics demonstrate that the proposed biosensor system is promising for solving the tasks of rapid monitoring.

### 2.3. Biosensor Validation with Real Water Samples

**Figure 5:**
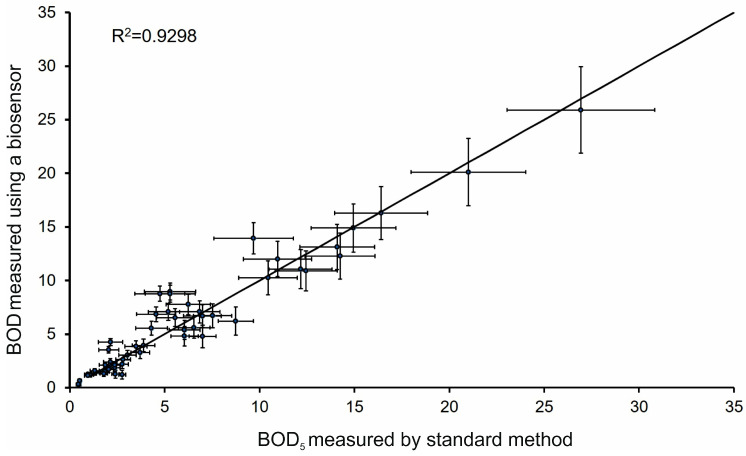
Correlation between BOD values measured by the developed biosensor and the standard BOD_5_ method for 53 environmental samples.

The biosensor analyzer “Expert-009” equipped with the developed receptor element was validated through a comparative analysis of 53 water samples collected from diverse water bodies in the Tula region, including small and medium rivers, lakes, reservoirs, and effluents from municipal treatment plants. A comparison of the BOD_5_ values obtained by the standard dilution method and via the biosensor based on *P. yeei* microorganisms in a xerogel matrix revealed a correlation between the methods, with a coefficient of R^2^ = 0.9298 (Figure 5). The regression line equation, y = 0.9461x + 0.4549, demonstrates an exceptionally high degree of agreement. A slope close to unity (0.9461) indicates a minimal systematic discrepancy, expressed as a slight underestimation of the biosensor readings by an average of 5.4% relative to the standard method. A small positive intercept (0.45 mg O_2_/dm^3^), observed in the low concentration range, may be explained by the background metabolic activity of the immobilized cells. The observed discrepancies are the resultant effect of counteracting factors. On one hand, processes such as kinetic limitations of substrate and oxygen diffusion through the xerogel matrix, as well as potential partial cell death during immobilization, may contribute to the underestimation of the biosensor signal. On the other hand, these trends are partially compensated by factors that could lead to an overestimation of results, such as the potential influence of electroactive compounds (heavy metal ions, sulfides, phenols) in real samples that oxidize on the electrode surface, as well as the higher metabolic activity of the immobilized *P. yeei* cells compared to the microbial consortium of the standard test. It is important to emphasize that, despite the complex nature of potential errors, their combined effect results in a minimal and statistically insignificant systematic deviation. The low magnitude of error, combined with the exceptional operational speed of the method (5 min vs. 5 days), confirms that the developed biosensor is a reliable tool for rapid screening and spatial monitoring of organic pollution.

The conducted comparative study (Table 2) demonstrates the competitiveness of the developed biosensor. Although the coefficient of determination (R^2^ = 0.929) is somewhat lower than in studies with a smaller sample size, the key advantages of our methodology are its exceptionally low systematic underestimation (−5.4%) compared to the overestimation (+13.9%) of an analogue, and its large-scale validation on 53 real samples compared to 8–9. This sample volume provides statistical power and reliability to the conclusions. Thus, the comprehensive analysis confirms the high accuracy and reliability of the biosensor for application in practical monitoring.

The research conducted demonstrates that the developed biosensor analyzer is a reliable tool for the rapid screening and spatial monitoring of organic pollution. The correlation with the standard method allows for its use in the operational assessment of water quality in field conditions, while the standard method remains relevant for precise laboratory measurements within the framework of regulatory control. To reduce systematic errors in samples with complex compositions, periodic calibration of the instrument using real water samples with a known BOD_5_ value is recommended.

### 2.4. Using Global Surface Water Maps in Combination with BOD_5_ Indicators to Identify Environmentally Critical Situations in the Region

**Figure 6:**
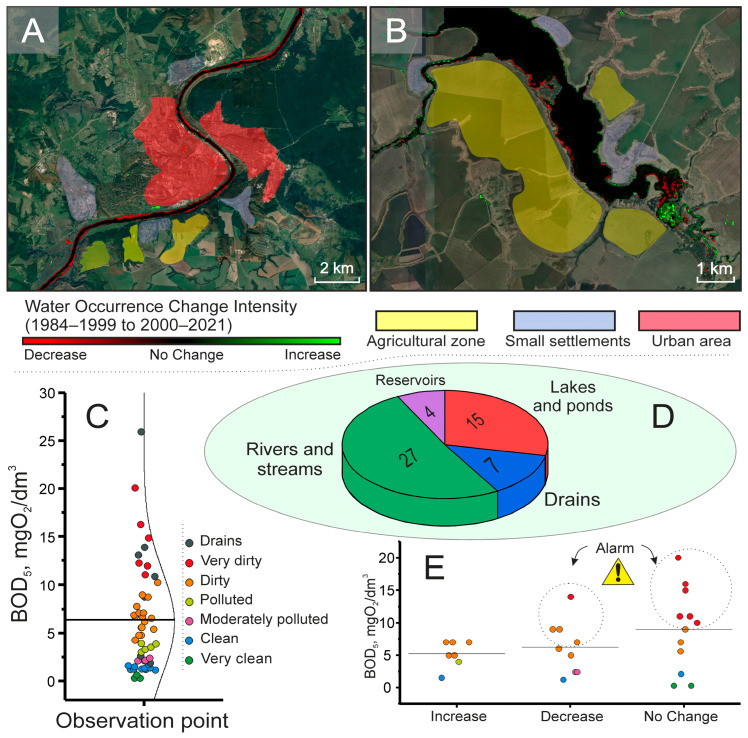
Integrated assessment of water quality in the Tula region. (**A**,**B**) Examples of river shoreline dynamics (reduction and stable/increasing). (**C**) Spatial distribution of BOD_5_ values from biosensor measurements. (**D**) Proportion of different water body types studied. (**E**) Statistics of shoreline changes and identification of pollution hotspots.

For the verification and spatial analysis of the data obtained with the biosensor, a comprehensive approach was applied, integrating the results of rapid BOD_5_ measurements with cartographic data from the Global Surface Water Explorer platform based on the Landsat satellite imagery archive. This synergic combination of methods enabled a transition from point-source pollution assessment to a systematic analysis of the ecological situation across the entire region. Figure 6 presents examples of this integrated approach to identifying the environmental status in a water body zone.

Based on the collected data, no obvious correlation was observed between the BOD_5_ value and changes in the shoreline, as high BOD_5_ values were present for water bodies with both increasing and decreasing shorelines (Figure 6). The most pronounced influence of a potential pollution factor on BOD was observed in reservoirs associated with effluents from treatment plants (Table 3). This correlation indicates inefficient treatment processes, necessitating enhanced oversight of their operation and potential modernization. A dependency was observed between the presence of industrial enterprises and BOD_5_ values. Water bodies located near enterprises operating in the food, livestock, and fish processing industries exhibited high BOD_5_ values. This phenomenon is likely caused by the discharge of effluents containing organic components, nutrients, and phosphorus- and nitrogen-containing compounds, which lead to water body eutrophication [47]. A similar situation was observed in Vietnam in a study of wastewater from livestock farms, which demonstrated a strong influence of industrial effluents on BOD values [48]. A moderate correlation with BOD_5_ values was found for the presence of nearby private settlements. This can be explained by the lack of centralized sewer systems, the intensive use of mineral and organic fertilizers, and improper management of plant waste. The strength of this factor’s influence depends on the local conditions of the water body: the type of terrain and soil, and the level of organization of the private settlements. Other factors demonstrating a weak or absent correlation with BOD_5_ included cemeteries and recreational areas around water bodies (recreation centers, beaches, parks). The potential influence of cemeteries could be due to the leaching of organic compounds by groundwater, which then enter the water body, causing pollution. Their impact is only possible if the cemetery is in immediate proximity, the groundwater level is high, and the cemetery area is prone to flooding. The influence of recreational zones is explained by anthropogenic pressure: the introduction of food waste, human waste, and the discharge of wastewater from infrastructure located within these areas into the water bodies.

Therefore, to prevent the occurrence of hazardous environmental situations in water bodies, primary consideration must be given to the close proximity of treatment plant effluents and industrial enterprises, as these anthropogenic factors demonstrate the most significant influence on organic water pollution. This is confirmed by the maximum BOD_5_ values recorded in areas of their location.

It is acknowledged that satellite remote sensing can be limited by cloud cover and spatial resolution. However, for this regional-scale study, the use of the long-term, aggregated data from the Global Surface Water Explorer platform mitigated these issues, providing a cloud-free, consistent dataset suitable for analyzing long-term hydrological changes in the water bodies of the Tula region.

Thus, satellite data serve as a powerful tool for initial screening, answering the question “Where should we look for a problem?” Meanwhile, the deployment of biosensor measurements provides precise quantitative verification, answering the question “How serious is the problem?” As a result of the study, it was established that the average regional BOD_5_ value was 6.5 mg O_2_/dm^3^ (Figure 6C), indicating a moderate level of organic pollution overall. However, the spatial distribution of the indicators was highly heterogeneous. The analysis revealed distinct ecological “hotspots”—localized areas with anomalously high BOD_5_ values that exceed the regional average threshold and correlate with data on shoreline reduction (Figure 6E). These zones most likely correlate with the location of point sources of anthropogenic impact, such as: wastewater discharges from municipal treatment plants, and industrial zones, particularly enterprises from the food and processing sectors. Consequently, the integration of remote sensing data and biosensor analysis represents not merely an addition of two methods, but a true synergy, where the capabilities of one compensate for the limitations of the other. Satellite monitoring provides broad coverage and identifies risk zones, while the portable BOD biosensor offers rapid and reliable quantitative confirmation of organic pollution directly in the field. The proposed approach lays the foundation for creating a cost-effective, highly efficient, and scalable environmental monitoring system at the regional level.

## 3. Conclusions

The conducted study demonstrates the successful implementation of an interdisciplinary approach, where advancements in materials science and biotechnology were integrated with modern remote sensing methods to address a pressing task of environmental monitoring. The key fundamental achievement of this work is the development and comprehensive characterization of a high-performance bioreceptor element based on *P. yeei* microorganisms immobilized in a xerogel matrix produced by sol–gel technology on a biocompatible PHB substrate. It was established that this immobilization system not only ensures the physical stabilization of the cells but also creates an optimal microenvironment that preserves their high metabolic activity, as confirmed by a suite of physicochemical analytical methods. This highlights the crucial importance of controlling material structure and properties at the micro-level in developing high-performance biosensing systems. From a practical standpoint, the developed biosensor demonstrated a correlation (R^2^ = 0.93) with the standard BOD_5_ method when tested on 53 real samples, confirming its accuracy and reliability for rapid analysis. However, the principal result lies not in the simple replacement of one method with another, but in the synergy achieved by combining biosensor monitoring data with long-term satellite observations. This integrated approach enabled a transition from point measurements to the systemic identification and ranking of anthropogenic impact sources on the region’s aquatic ecosystems, revealing organic pollution “hotspots”. Future prospects of the work involve the further optimization of the xerogel matrix structure to increase the biosensor’s operational lifetime, as well as the development of automated remote systems based on it. A network of such biosensors—combined with stationary or mobile sensors and continuous satellite monitoring—can form the foundation for creating fundamentally new, cost-effective, and highly efficient environmental control systems operating in near-real time. This application represents a critically important starting point on the path toward sustainable water resource management.

## 4. Materials and Methods

### 4.1. Cultivation of Microorganisms

Two microbial strains were used in this work: *Paracoccus yeei* and *Cupriavidus necator* VKM B-3386. *P. yeei*, previously isolated from the activated sludge of a municipal treatment plant and demonstrated to be effective for BOD analysis, was selected as the bioreceptor for the BOD biosensor. The *Cupriavidus necator* VKM B-3386 strain, obtained from the All-Russian Collection of Microorganisms (VKM), was used to synthesize PHB intended for use as a substrate. Both cultures were maintained on Petri dishes with agarized LB medium of the following composition (g/dm^3^): tryptone—5, yeast extract—10, NaCl—10, bacteriological agar—20.

#### 4.1.1. Separation of *P. yeei* Bacterial Biomass

To accumulate biomass, the strain was cultivated in liquid LB medium. Erlenmeyer flasks containing 150 mL of the medium were sterilized by autoclaving at 120 °C for 30 min. Inoculation was performed aseptically in a laminar flow hood by transferring biomass from a fresh agarized medium. Cultivation was carried out in a shaker incubator at 28 °C and 180 rpm for 72 h. Cells were harvested by centrifugation at 8000 rpm for 10 min at room temperature. The pellet was washed twice with 20 mM potassium-sodium phosphate buffer (pH = 6.8) and centrifuged again under the same conditions. The obtained biomass was weighed, resuspended in a small volume of the same buffer to a concentration of 150 mg/cm^3^, and stored in Eppendorf-type tubes at 5 °C until further use for biosensor creation.

#### 4.1.2. Cultivation of *Cupriavidus necator* VKM B-3386, PHB Extraction, and Film Fabrication

The strain was cultivated in a nitrogen-limited mineral medium for maximum intracellular accumulation of PHB. The medium composition was (g/dm^3^): glucose—20, Na_2_HPO_4_·12H_2_O—9, KH_2_PO_4_—1.5, NH_4_Cl—1, MgSO_4_·7H_2_O—0.2, CaCl_2_—0.015. The medium was sterilized by autoclaving at 1.15 atm for 45 min. Cells were incubated under aerobic conditions at 28 °C with shaking at 180 rpm for 96 h. After incubation, cells were harvested by centrifugation (8000 rpm, 10 min).

Briefly, the microbial pellet was dried and then treated with chloroform at 50 °C for 2 h, with periodic sonication in an ultrasonic bath. The polymer was then precipitated by adding an excess of hexane on an ice bath. The obtained PHB was purified by evaporation using a vacuum rotary evaporator. The polymer was dried, and 1.5 g of PHB was dissolved in 25 mL of chloroform (resulting in a 6% *w*/*v* solution) and cast into glass Petri dishes with a diameter of 95 mm. The films were allowed to dry under ambient conditions for 2 h at 30–40 °C to facilitate controlled solvent evaporation and pore formation, resulting in free-standing films with a final thickness of 80 ± 10 μm. The surface porosity was an intrinsic characteristic of this solvent casting process and was not subjected to additional post-treatment. The resulting films were cut into specified fragments (5 mm × 5 mm) for further use.

### 4.2. The Formation of a Bioreceptor Element Using a Xerogel Created Through Sol–Gel Technology on a Poly(3-hydroxybutyrate) Substrate

**Figure 7:**
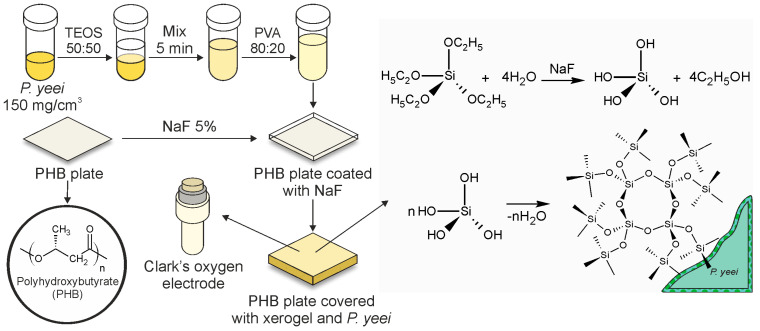
Schematic illustration of the bioreceptor element fabrication process.

To form the bioreceptor element (Figure 7), a potassium-sodium phosphate buffer (pH = 6.8) was added to the biomass of *P. yeei* microorganisms to achieve a cell titer of 150 mg/cm^3^. The resulting mixture was vortexed for five minutes. A tetraethoxysilane (TEOS) solution was added to the prepared microbial suspension in a 50:50 vol% ratio and mixed again. A polyvinyl alcohol (PVA) solution was then introduced into the resulting suspension in an 80:20 vol% ratio, respectively. The PHB film substrate was moistened with a 5% sodium fluoride (NaF) solution. Then, 20 µL of the prepared suspension described above was applied to the moistened surface. After applying the suspension with microorganisms onto the PHB substrate, it was dried and subsequently attached to the working surface of the dissolved oxygen sensor (oxygen electrode).

### 4.3. Calibration of the Dissolved Oxygen Sensor

Prior to applying the bioreceptor element, the oxygen electrode required preparation according to the manufacturer’s instructions. This involved calibrating the instrument by setting the current values in the device’s settings menu for both a zero-oxygen environment and an environment with the maximum saturated concentration of dissolved oxygen in water. Sodium sulfite (Na_2_SO_3_) was added to the water to remove oxygen, while maximum oxygen solubility was achieved by purging with air using a pump for 5 h.

### 4.4. Scanning Electron Microscopy with Energy Dispersive X-Ray Analysis (SEM-EDX)

The surface morphology of the investigated samples was analyzed using a Hitachi TM 4000 Plus scanning electron microscope (Hitachi High-Tech Corporation, Tokyo, Japan). Images were acquired in either secondary electron (SE) or backscattered electron (BSE) mode at an accelerating voltage of 15 kV. SEM combined with EDX analysis was performed using an EDX attachment (Bruker, Karlsruhe, Germany) at an accelerating voltage of 20 kV.

### 4.5. Raman Spectroscopy

Sample analysis was conducted using a Photon-Bio M532 instrument (Spektr-M, Chernogolovka, Moscow, Russia). Each spectrum was recorded at least 7 times from 3 different points on the sample. The exposure time was 8000 ms, and the number of accumulation cycles was 10. The wavelength of the laser excitation source was 532 nm.

### 4.6. IR Spectroscopy

Sample analysis was performed using an InfraLUM FT-08 FT-IR spectrometer (Lumex, Saint Petersburg, Russia). Absorption spectra were recorded in the range of 4000–500 cm^−1^. Samples were registered both as fixed polymer films and as pellets mixed with KBr.

### 4.7. NMR Spectroscopy of PHB Films

The nuclear magnetic resonance spectra (^1^H and ^13^C NMR) of PHB were recorded on a Bruker Avance III HD Fourier 300 spectrometer (operating frequency of 300 MHz for ^1^H, Bruker Corporation, Visp, Switzerland). Measurements were carried out in a solution of deuterated chloroform (CDCl_3_). Chemical shifts (δ, ppm) were calibrated relative to the signal of the residual proton of CDCl_3_ (δ = 7.26 ppm for ^1^H) and the central signal of the CDCl_3_ triplet (δ = 77.16 ppm for ^13^C).

### 4.8. Investigation of the Thermal and Physicomechanical Properties of the Produced PHB Film

The thermal behavior of the biopolymer was investigated by simultaneous thermogravimetric analysis and differential scanning calorimetry (TGA-DSC) using a synchronous analyzer SKZ1053A (SKZ Industrial Co., Limited, Jinan, China). A sample of the PHB film produced by *C. necator* VKM B-3386, weighing 11.35 mg, was heated in an inert nitrogen atmosphere in the range of 25–800 °C at a heating rate of 10 °C/min. During a single experiment, the change in sample mass (TGA) and heat flow (DSC) were recorded simultaneously.

The physico-mechanical properties of the PHB film were determined in accordance with the methodology described in the Supplementary Materials.

### 4.9. Objects of Environmental Research

The study was conducted on small (length < 50 km, catchment area < 500 km^2^), medium (50–200 km, 500–2000 km^2^), and large (>200 km, >2000 km^2^) watercourses in the Tula region, belonging to the basins of the Oka and Don rivers. The selection criteria were: the degree of anthropogenic load (industrial enterprises, agricultural areas, urbanized zones), hydrological significance (contribution to the flow formation of major river systems), and accessibility for field measurements. Additionally, artificial ponds and lakes of various purposes (recreational, fishery, technical) were studied, as well as discharges from municipal treatment plants performing biological wastewater treatment. For each site, control sampling points were defined. Water sampling was carried out over the month of July during stable hydrological conditions (no precipitation > 48 h, average daily air temperature 18–21 °C). A standardized protocol was used: surface samples (0–30 cm depth) were collected into sterile dark glass bottles, immediately cooled to 4 °C, and promptly delivered to the laboratory.

To ensure analytical reproducibility, turbid samples containing suspended solids and phytoplankton were filtered through 7–20 μm membrane filters, while clear samples were analyzed without pretreatment. For the standard BOD_5_ determination, samples with expected high organic load were appropriately diluted (typically 2–4 fold) with phosphate buffer (pH 6.8) according to standard methodology requirements. The measured BOD_5_ values covered a broad spectrum from 0.5 to 27 mg O_2_/dm^3^, encompassing various pollution levels—from conditionally clean (BOD_5_ < 2 mg O_2_/dm^3^) to heavily polluted (BOD_5_ > 15 mg O_2_/dm^3^) waters according to standard water quality classifications.

### 4.10. The Standard Method for Determining the BOD_5_ Indicator

For BOD analysis, the standard method and the biosensor-based approach, described in detail in the following sections, were applied. For comparison of the results and further validation of the BOD biosensor, the standard dilution method for BOD determination was used. Analysis of the collected water samples was performed according to the methodology specified in [49]. The dissolved oxygen content was determined using an EXPERT-001-4.0.1 BOD thermo-oximeter (Econix-Expert LLC, Moscow, Russia).

### 4.11. Biosensory Measurements

A 4.0 mL volume of potassium-sodium phosphate-buffered solution (pH = 6.8) was introduced into a measurement cell (a 5 mL chemical beaker). After establishing a constant oxygen concentration (a stable baseline), a GGA solution (glucose-glutamic acid mixture with a concentration of 300 mg/L) was added in volumes ranging from 2 µL to 200 µL. During the measurements, the dependence of dissolved oxygen concentration (in mg/dm^3^) on measurement time was displayed on the computer monitor. The biosensor response was recorded as the rate of change in the dissolved oxygen concentration upon the addition of the GGA solution. Mathematically, the response was defined as the tangent of the slope angle of the linear approximating curve to the abscissa axis, equivalent to the coefficient ‘a’ for ‘x’ in the equation of the calibration curve (y = ax + b). The obtained results were processed using computer software.

### 4.12. Analyzing Changes in the Coastline and Identifying Potential Sources of Pollution

The Global Surface Water Explorer platform, which provides maps showing the location and temporal distribution of water surfaces on a global scale, was used to determine shoreline changes. The water body corresponding to the sampling point coordinates was localized on the map. Based on applying a filter for water occurrence change intensity, a conclusion was made regarding the increase, decrease, or absence of shoreline changes. The nearest potential pollution sources for the water bodies were identified using topographic maps of the area and openly available data on the internet.

The integration of satellite and biosensor data was performed through a two-stage geospatial analysis. First, potential pollution risk zones were identified based on the hydrological change intensity layers from the Global Surface Water Explorer. Subsequently, these pre-selected zones were ground-truthed using the BOD biosensor. The final spatial assessment and ranking of pollution sources were conducted by overlaying the precise BOD measurement points onto the map of anthropogenic factors and observed hydrological changes, confirming the correlation qualitatively and statistically without the use of a predictive machine learning model.

## Figures and Tables

**Table 1:** Main analytical and metrological characteristics of the developed biosensor and its analogs.

Parameter	This Work	[45]	[15]	[45]	[46]
Range of Determined Concentrations, mgO_2_/dm^3^	0.5–50	0.05–5.0	10–220	0.34–9.6	0.1–21
Time of a single biosensor measurement, min	~5	4–6	20	30–130	4–6
RSD, %	<15	7	3.1	4.1	7
Long-term stability, days	20	45	-	7	11
Number of oxidizable substrates	22 of 25	32 of 33	-	12 of 14	20 of 24

**Table 2:** Comparison of real sample analysis results obtained in this study with literature data.

Parameter	This Work	[44]	[15]	[46]
The sensitive element	*P. yeei* in a xerogel matrix on PHB	*P. yeei* in modified PVA hydrogel	*S. cerevisiae* on bacterial cellulose	*P. yeei* in a xerogel matrix
R^2^	0.929	0.999	0.986	0.998
Overestimation or underestimation of biosensor parameters compared to the standard method for measuring BOD, %.	Underestimation by 5.4%	-	Overestimation by 13.9%	-
The number of real samples examined	53	9	8	9

**Table 3:** Ranking of factors influencing BOD_5_ values.

Rank	The Influence Factor	The Magnitude of the Impact on BOD
1	Wastewater treatment plants	Very high
2	Industrial enterprises (food, livestock)	High
3	Private settlements	Moderate/Average
4	Recreational areas (beaches, recreation centers, parks)	Weak
5	Graveyard	Weak/Background

## Data Availability

The data that support the findings of this study are available on request from the corresponding author.

## Supplementary Materials

The following supporting information can be downloaded at: https://www.mdpi.com/article/10.3390/gels11110849/s1. Table S1. Physico-mechanical characteristics of the PHB film [50]; Table S2: Absorption frequencies in the Raman spectrum of the PHB [51]; Table S3: Absorption frequencies in the FTIR spectrum of the PHB [52]; Table S4: Elemental composition of the PHB film; Table S5: Elemental composition of *P. yeei* microorganisms coated with a xerogel matrix on a PHB substrate; Table S6: Elemental composition of *P. yeei* microorganisms coated with a xerogel matrix extracted from a PHB substrate; Figure S1. Graph of the dependence of the load on displacement, obtained by stretching the PHB film; Figure S2. ^13^C NMR spectrum of synthesized PHB [53]; Figure S3. A ^1^H NMR spectrum of synthesized PHB. TGA-DSC of synthesized PHB [54,55]; Figures S4–S8. Energy dispersive X-ray spectroscopy of the PHB film; Figures S9–S13. Energy-dispersive X-ray spectroscopy of *P. yeei* microorganisms coated with a xerogel matrix on a PHB substrate; Figures S14–S18. Energy-dispersive X-ray spectroscopy of *P. yeei* microorganisms coated with a xerogel matrix extracted from a PHB substrate. Figures S19–S32. Scanning electron microscopy of a PHB film with *P.yeei* microorganisms adsorbed on it, coated with a xerogel matrix. Figures S33–S46. Scanning electron microscopy of a dried bioreceptor element formed in the work. Figure S47: Long-term stability of the formed biosensor; Figures S48–S51. Example of biosensor operation.
