# Supplementary material for: A Polyhydroxybutyrate-Supported Xerogel Biosensor for Rapid BOD Mapping and Integration with Satellite Data for Regional Water Quality Assessment
Source: Gels. 2025 Oct 24;11(11):849. doi: 10.3390/gels11110849 (PMC12651753; doi:10.3390/gels11110849)
Supplement: Supplementary file 1 [file gels-11-00849-s001.zip › gels-3925455-supplementary.pdf]

Supporting Information for the article:

A Polyhydroxybutyrate-Supported Xerogel Biosensor for Rapid BOD Mapping and Integration with Satellite Data for Regional Water Quality Assessment

George Gurkin[1], Alexey Efremov[1], Irina Koryakina[1], Roman Perchikov[1], Anna Kharkova[1], Anastasia Medvedeva[1], Bruno Fabiano[2], Andrea Pietro Reverberi[3], Vyacheslav Arlyapov[1*].

[1] BioChemTech Research Center, Tula State University, pr. Lenina 92, 300012 Tula, Russia;
[2] DICCA – Civil, Chemical and Environmental Engineering Department – Genoa University, Via Opera Pia, 15 – 16145 Genoa, Italy;
[3] DCCI—Department of Chemistry and Industrial Chemistry, Genoa University, Via Dodecaneso 31, 16146 Genoa, Italy;

*E-mail: v.a.arlyapov@tsu.tula.ru

Table of contents

1. Determination of the physical and mechanical properties of the PHB film 2
2. Results determination of the physico-mechanical properties of the PHB film 3
3. Characterization of the PHB film by Raman spectroscopy 5
4. Characteristics of the PHB film by IR spectroscopy 6
5. NMR spectroscopy of synthesized PHB 7
6. Thermogravimetric analysis and differential scanning calorimetry (TGA-DSC) of synthesized PHB 10
7. Energy dispersive X-ray spectroscopy of the PHB film 12
8. Energy-dispersive X-ray spectroscopy of *P. yeei* microorganisms coated with a xerogel matrix on a PHB substrate 15
9. Energy-dispersive X-ray spectroscopy of *P. yeei* microorganisms coated with a xerogel matrix extracted from a PHB substrate 18
10. Scanning electron microscopy of a PHB film with *P.yeei* microorganisms adsorbed on it, coated with a xerogel matrix 22
11. Scanning electron microscopy of a dried bioreceptor element formed in the work 29
12. Long-term stability of the biosensor 37
13. Correction factor for enhanced BOD quantification 38
14. Quantitative analysis of diffusion limitations 39
15. Example of biosensor operation 40
Reference 42

1. Determination of the physical and mechanical properties of the PHB film

Before the test, the thickness and width of the sample were measured in three places. The sample was placed in special clamps that were attached to the grips of the testing machine. The clamps were designed to ensure that the sample was securely fastened and that the longitudinal axis of the sample aligned with the direction of stretching. The sample was then loaded, and the loading rate was chosen to ensure that the time from the application of the load to the sample's failure was at least 60 seconds for materials with a yield strength and at least 30 seconds for materials without a yield strength. The following parameters are determined from the obtained stretching diagrams:

- – tensile strength, $\sigma_{\text{рм}}$, MPa

$$\sigma_{\text{рм}} = \frac{F_{\text{рм}}}{\mathrm{A}_0}; \tag{1}$$

- – tear strength, $\sigma_{\text{рр}}$, MPa

$$\sigma_{\text{рр}} = \frac{F_{\text{рр}}}{\mathrm{A}_0}; \tag{2}$$

- – tensile yield strength, $\sigma_{\text{рт}}$, MPa

$$\sigma_{\text{рт}} = \frac{F_{\text{рт}}}{\mathrm{A}_0}; \tag{3}$$

- – conditional tensile yield strength, $\sigma_{\text{рту}}$, MPa

$$\sigma_{\text{рту}} = \frac{F_{\text{рту}}}{\mathrm{A}_0}; \tag{4}$$

Where $F_{\text{рм}}$ — maximum tensile load, N;

$F_{\text{рр}}$ — the tensile load at which the sample collapsed, N;

$F_{\text{рт}}$ — tensile load when the yield strength is reached, N;

$F_{\text{рту}}$ — tensile load when the conditional yield strength is reached, N;

A_0 — the initial cross-section of the sample, mm^2.

– elongation at maximum voltage (load), $\varepsilon_{\text{рм}}$, %

$$\varepsilon_{\text{рм}} = \frac{\Delta l_{\text{ом}}}{L_0} 100; \tag{5}$$

– – elongation at break, ε_{pp}, %

$$\varepsilon_{\text{pp}} = \frac{\Delta l_{\text{op}}}{L_0} 100; \quad (6)$$

– – elongation at yield strength, $\varepsilon_{\text{pт}}$, %

$$\varepsilon_{\text{pт}} = \frac{\Delta l_{\text{oт}}}{L_0} 100; \quad (7)$$

Where $\Delta l_{\text{oм}}$ — change in the calculated length of the sample at the moment when the maximum tensile load is reached, mm;

L_0 — initial estimated sample length, mm;

Δl_{op} — change in the estimated length of the sample at the moment of rupture, mm;

$\Delta l_{\text{oт}}$ — change in the estimated length of the sample at the moment of reaching the yield point, mm.

2. Results determination of the physico-mechanical properties of the PHB film

The deformation and strength characteristics, namely ultimate strength, yield strength, elongation, and Young's modulus, were determined for the obtained PHB films. The resulting deformation curve is shown in Figure S1.

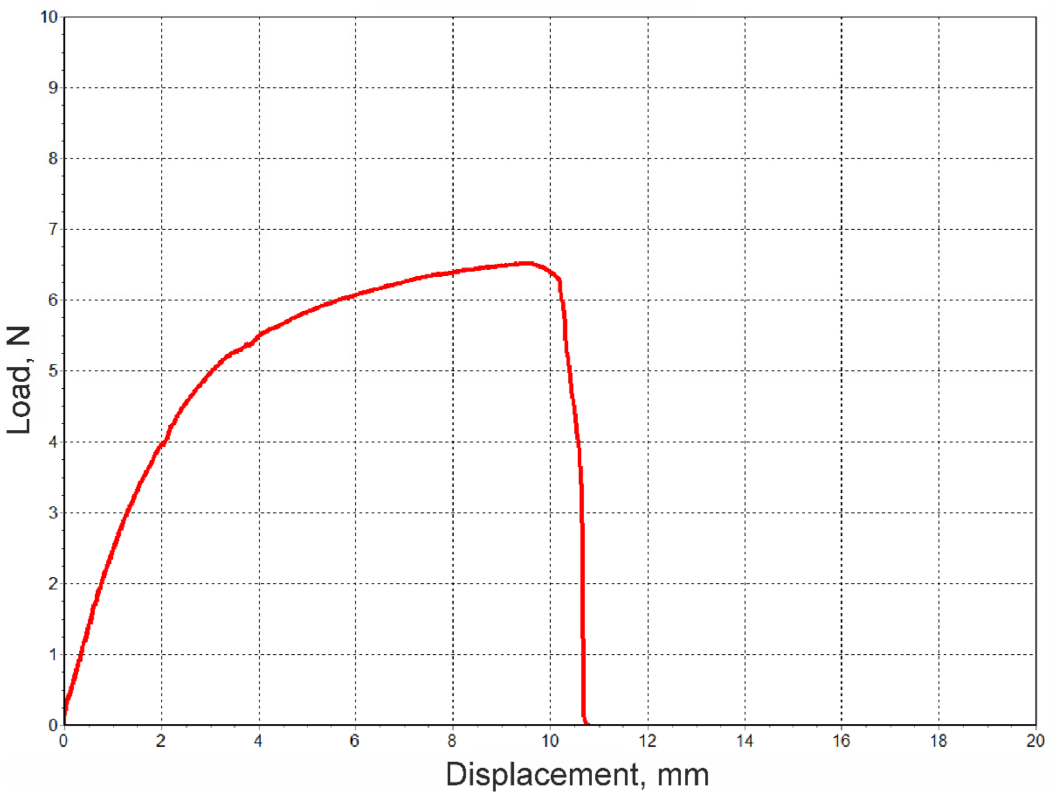

Figure S1. Graph of the dependence of the load on displacement, obtained by stretching the PHB film

The deformation and strength characteristics were calculated according to the protocol and in the Bluehill automated program. The data obtained are presented in Table S1.

Table S1 — Physico-mechanical characteristics of the PHB film

Characteristic	Experimental data	Literary data
Tensile strength, MPa	6.5±0.5	5-12
Conditional yield strength, MPa	4.5±0.9	-
Relative elongation at max load, %	12±4	-
Relative elongation at break, %	15±4	6
Bearing capacity of the material, N/m	540±30	-
Young's modulus, MPa	250±10	>200

The mechanical properties of the studied PHB film (tensile strength 6.5±0.5 MPa, Young's modulus 250±10 MPa) correspond to the range of values for undirected samples obtained by casting from solution. Such films are characterized by a strength of 5-12 MPa and an elastic modulus of 200-1200 MPa, which is explained by the low degree of crystallinity and the absence of material extraction during the molding process [1].

The elongation at break (15%) indicates a moderate plasticity of the film, which is typical for PHB. The bearing capacity of the material (540 N/m) and the conditional yield strength (4.5 MPa) additionally confirm that the film has balanced mechanical properties combining strength and ductility. Thus, the obtained physico-mechanical characteristics of the PHB film correspond to the data presented in the literature and indicate its suitability for use as a biodegradable material with satisfactory strength and deformation characteristics. In the future, it is possible to improve mechanical performance by optimizing the conditions of film formation and increasing the degree of crystallinity.

3. Characterization of the PHB film by Raman spectroscopy

When analyzing the obtained spectrum, it was found that the band at 2932 cm^{-1} can be attributed to the stretching of CH. The absorption bands observed at 1726 cm^{-1} and 1056 cm^{-1} correspond to carbonyl (C=O) stretching of the ester group and the C–O group, respectively. These two bands are identified as PHB signals. The absorption bands at 1443 and 1365 $^{-1}$ correspond to the asymmetric and symmetric deformation of the $–CH_3$ groups, respectively. The absorption band at 1101 cm^{-1} corresponds to the stretching of the C–O–C group, and at 839 cm^{-1} corresponds to the stretching of C–COO. The obtained absorption bands are consistent with the literature values of PHB Raman spectroscopy (Table S2) [2].

Table S2 shows the absorption frequencies, according to the literature data, and the results obtained on Photon-Bio M532.

Table S2 — Absorption frequencies in the Raman spectrum of the PHB

Characteristic group	The Raman shift, cm^{-1}	
	Lit. data	Exp. data
CH stretching	2883	2932
C=O stretching	1725	1726
CH_3 is asymmetric. deformation	1443	1443
CH_3 simm. deformation	1365	1365
C–O–C simm. stretching	1101	1101
C–O stretching	1058	1056
C–COO stretching	841	839

4. Characteristics of the PHB film by IR spectroscopy

The IR spectroscopy method was used to confirm the formation of PHB in *C. necator* VKM-3386 microorganisms. When analyzing the obtained spectrum, it was found that the absorption band at 3436 cm^{-1} corresponds to the stretching of the hydroxyl group (−OH). The bands observed in the 2930 cm^{-1} spectral region correspond to the stretching of the CH bond characteristic of PHB polymers. The absorption bands at 1725 cm^{-1} and 1279 cm^{-1} correspond to carbonyl (C=O) stretching of the ester group and the C–O group, respectively. These two bands serve as indicators of the presence of PHB. The absorption bands at 1455 and 1379 cm^{-1} correspond to the asymmetric and symmetric deformation of the $-CH_3$ groups, respectively. The absorption band at 1057 cm^{-1} corresponds to the stretching of the C–O–C group. The obtained absorption bands are consistent with the literature values of the PHB IR Fourier spectroscopy [3] (Table S3)

Table S3 shows the absorption frequencies, according to the literature data, and the results obtained on the infrared Fourier spectrometer Infralume FT-08.

Table S3 — Absorption frequencies in the FTIR spectrum of the PHB

Characteristic group	Wave number, cm $^{-1}$	
	Lit. data	Exp. data
OH stretching	3446	3436
CH stretching	2900	2930
C=O stretching	1720	1725
CH_3 is asymmetric. deformation	1453	1455
CH_3 simm. deformation	1380	1379
C–O stretching	1283	1279
C–O–C stretching	1045	1057

5. NMR spectroscopy of synthesized PHB

Nuclear magnetic resonance spectroscopy was used to study the structure of the polymer synthesized by *C. necator* VKM-3386 microorganisms. The resulting ^{13}C NMR spectrum is shown in Figure S2.

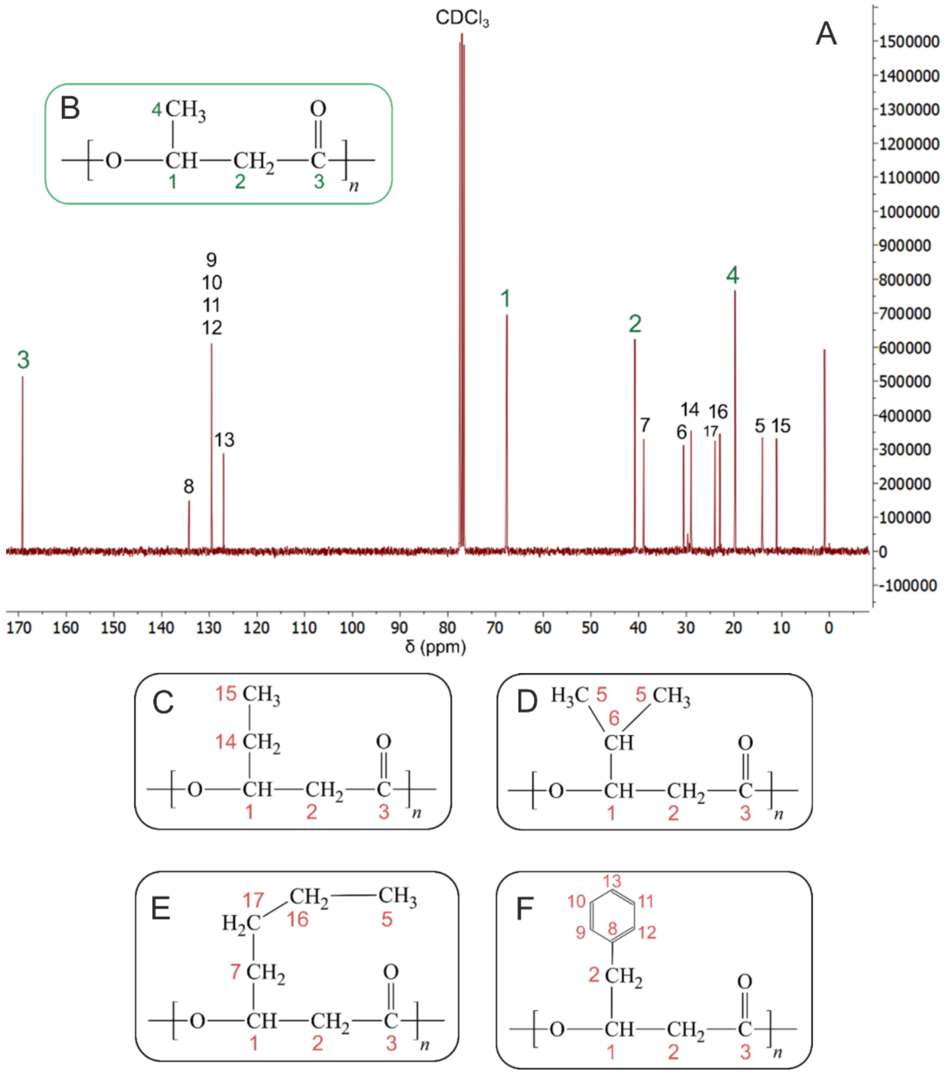

Figure S2. A — ^{13}C NMR spectrum of synthesized PHB; interpretation of the spectrum for: B — polyhydroxybutyrate; C — (poly(3-hydroxyvalerate); D — poly(3-hydroxy-4-methylvalerate); E — poly(3-hydroxyoctanoate); F — poly(3-hydroxy-5-phenylbutyrate)

During the decoding of the spectrum, it was found that the most intense signals in the 170, 68, 42, and 20 ppm regions correspond to atomic nuclei in the PHB [4]. Additional signals are also noted on this spectrum, which, when analyzing the literature data, were interpreted as other PHAs (poly(3-hydroxyvalerate), poly(3-hydroxy-4-methylvalerate), poly(3-hydroxyoctanoate), poly(3-hydroxy-5-phenylbutyrate)), which are part of the polymer. It is known that these PHAs are also synthesized by microorganisms and form copolymers with PHB. When decoding the

NMR spectrum, it was found that the units of the above-described PHAs can also be part of the resulting polymer.

The data obtained are also consistent with the ^{1}H NMR spectrum for the isolated polymer (Figure S3). For PHB, there is a multiplet in the region of 5.25 ppm, characteristic of the CH group, a doublet in the region of 1.3 ppm, for the CH3 group, and a signal in the region of 2.5 ppm, corresponding to the CH_2 group. The data obtained are consistent with the literature data for all proposed compounds and do not contradict the proposed structure. For the remaining GHAS, the interpretation is based on the spectrum and formulas. There is also a peak in the range of 0.06 ppm, which indicates the presence of polydimethylsiloxane. Its occurrence is associated with the ingress of vacuum lubricant into the sample during the extraction and separation of polymer on a rotary evaporator. A similar signal associated with this compound is observed on the ^{13}C NMR spectrum.

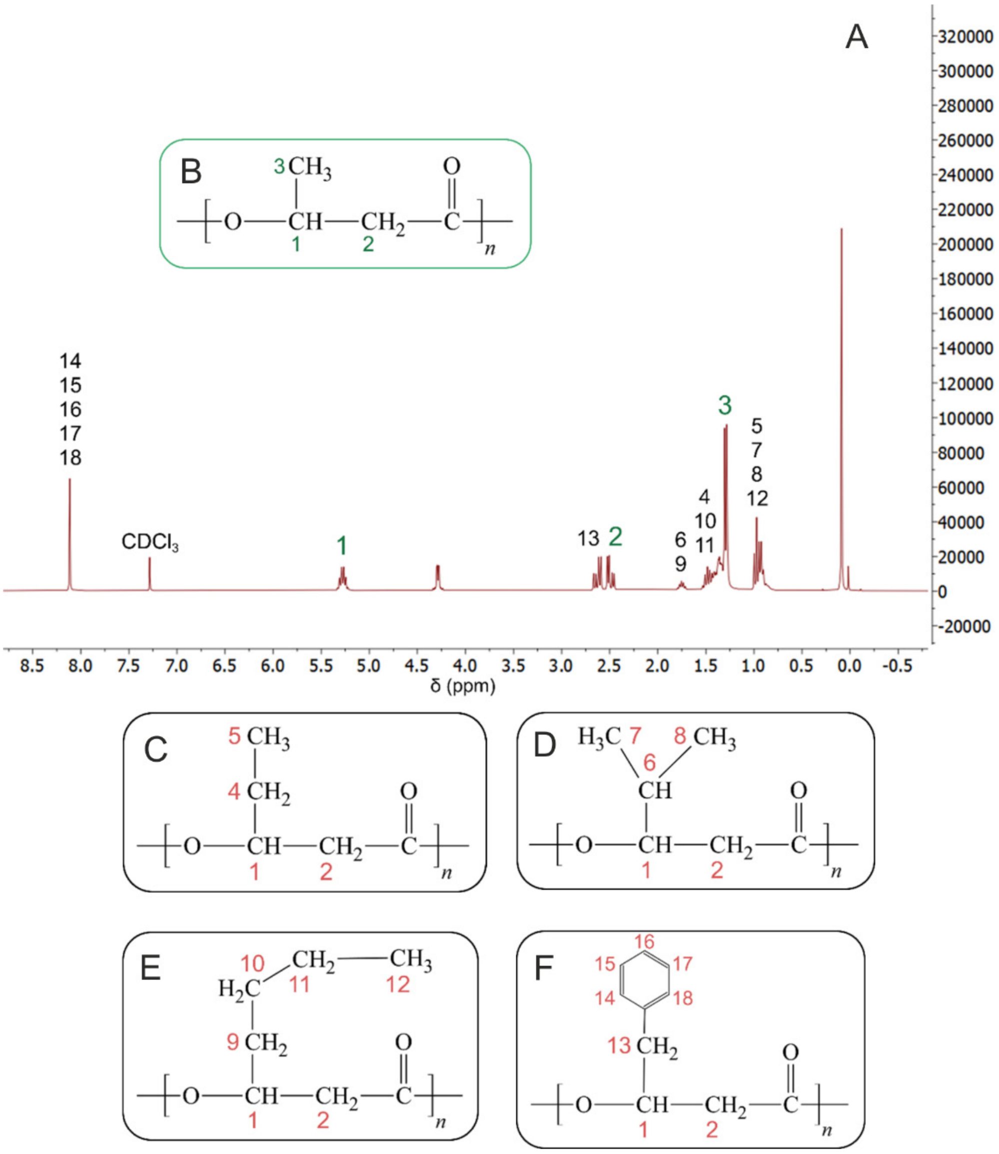

Figure S3. A — ^{1}H NMR spectrum of synthesized PHB; interpretation of the spectrum for: B — polyhydroxybutyrate; C — (poly(3-hydroxyvalerate); D — poly(3-hydroxy-4-methylvalerate); E — poly(3-hydroxyoctanoate); F — poly(3-hydroxy-5-phenylbutyrate)

6. Thermogravimetric analysis and differential scanning calorimetry (TGA-DSC) of synthesized PHB

The method of thermogravimetric analysis and differential scanning calorimetry (TGA-DSC) was used to study the thermal stability of the biopolymer. The analysis of the DSC curve revealed a peak corresponding to the melting process (Tm = 172.9°C) and the most pronounced endothermic peak characterizing the process of thermal decomposition of PHB (Td = 302.7°C). At the same time, the

biopolymer began to lose mass from 240 °C, and the maximum decomposition rate was reached at ~300°C.

The TG-DSC results characterized the thermal stability of the analyzed biopolymer, revealing a significant interval between the melting point (172.9°C) and the decomposition temperature (302.7°C). This is extremely important, since one of the main problems of industrial application of biopolymers is that the melting and decomposition temperatures of many of them are located very close to each other, which limits their manufacturability due to thermal decomposition in extrusion and injection molding processes [5].

The observed melting point (~177 °C) and high thermal stability (up to 300 °C) are consistent with literature data for PHB and are characteristic of a crystalline biopolymer [6].

7. Energy dispersive X-ray spectroscopy of the PHB film

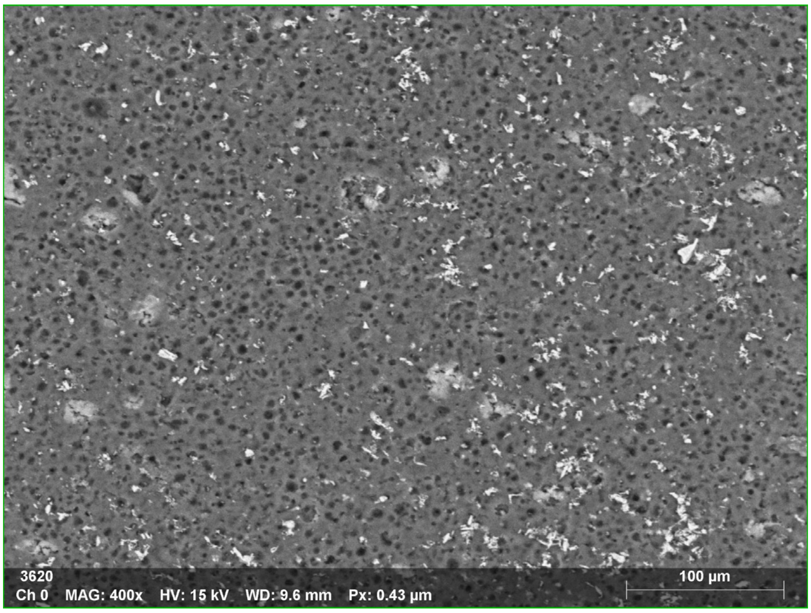

Figure S4. SEM-image of a PHB film, bar label 100 microns

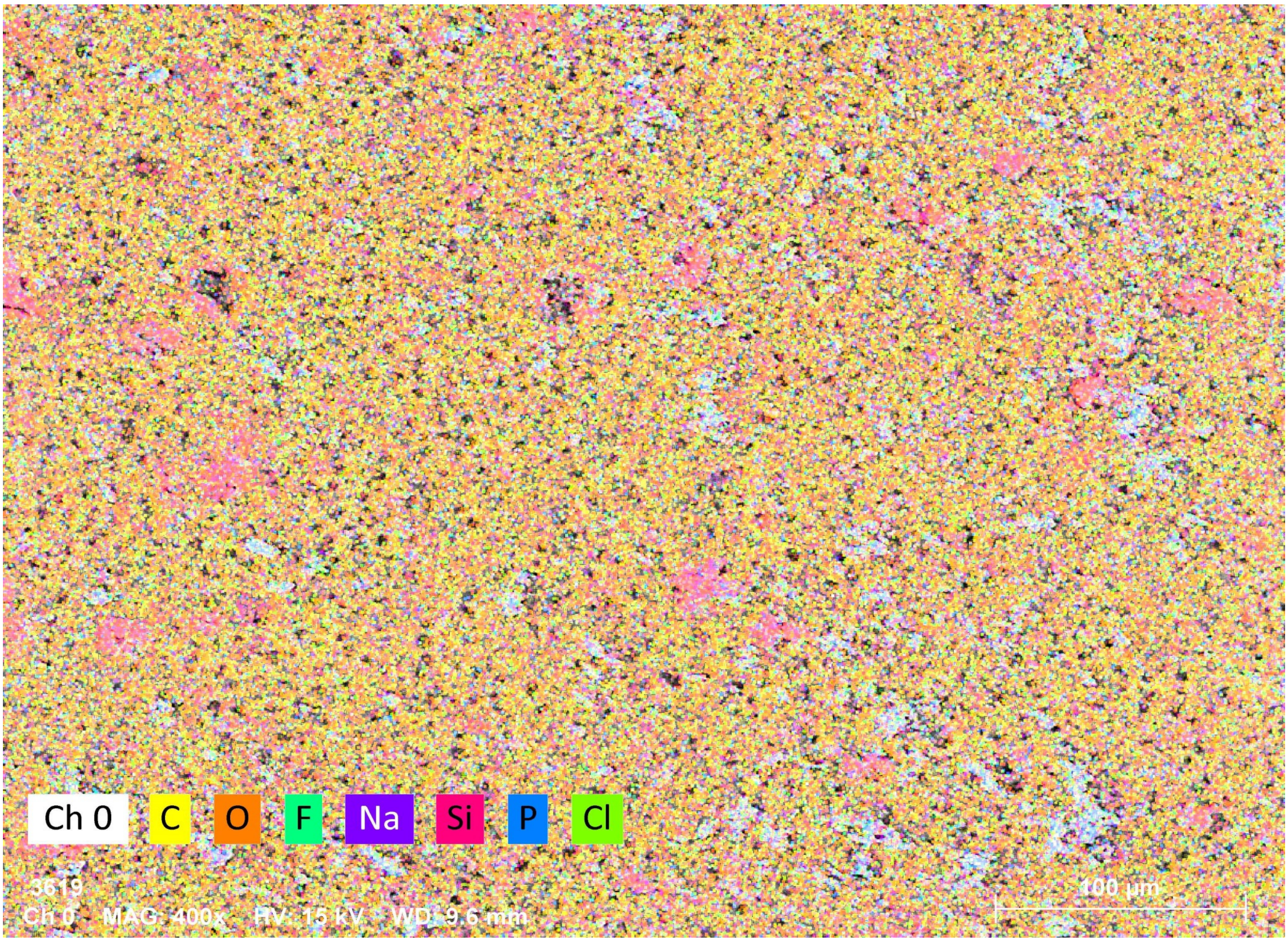

Figure S5. Map of the elements of the PHB film, bar label 100 microns

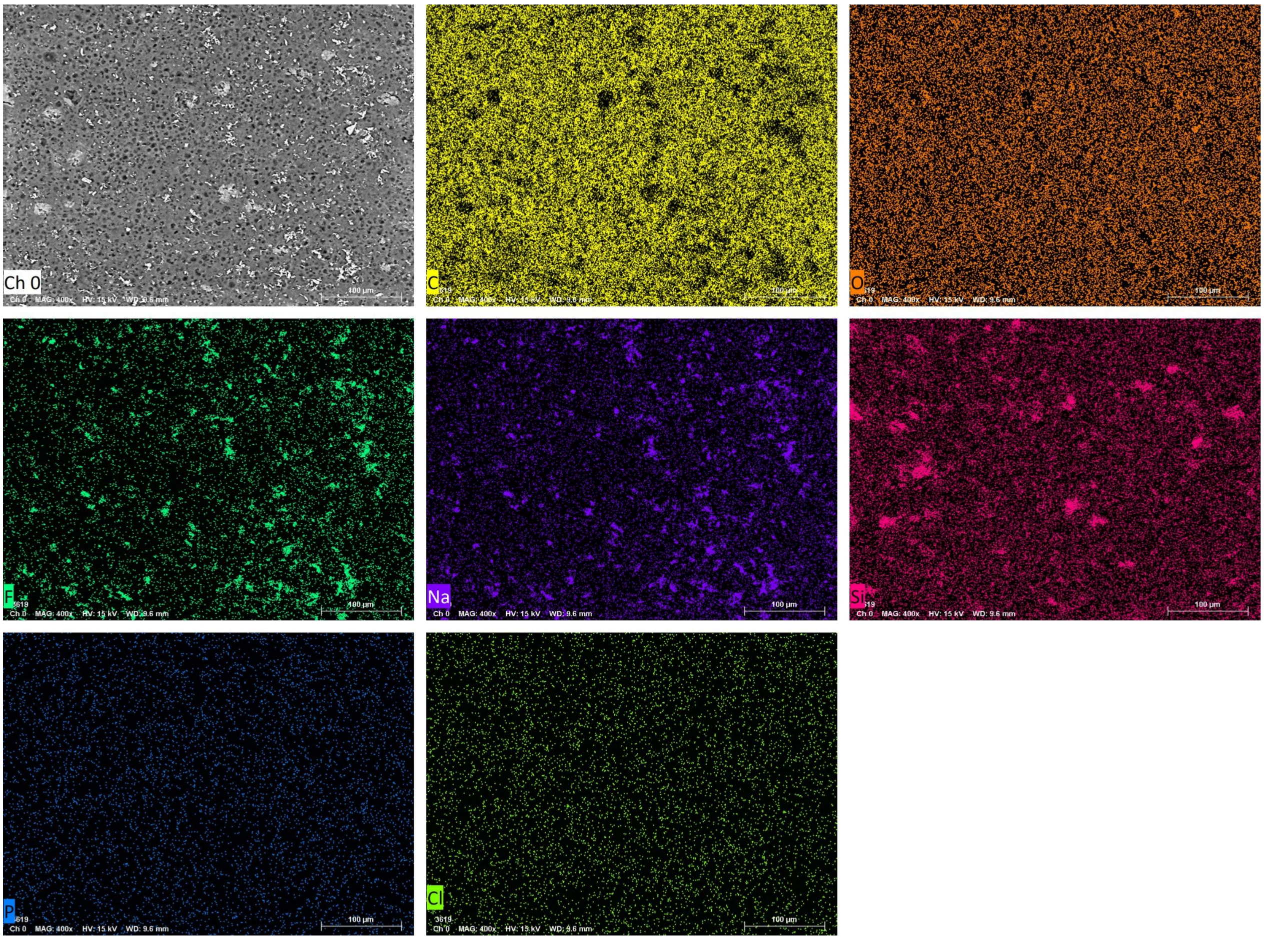

Figure S6. Distribution maps of each of the elements of the PHB film, bar label 100 microns

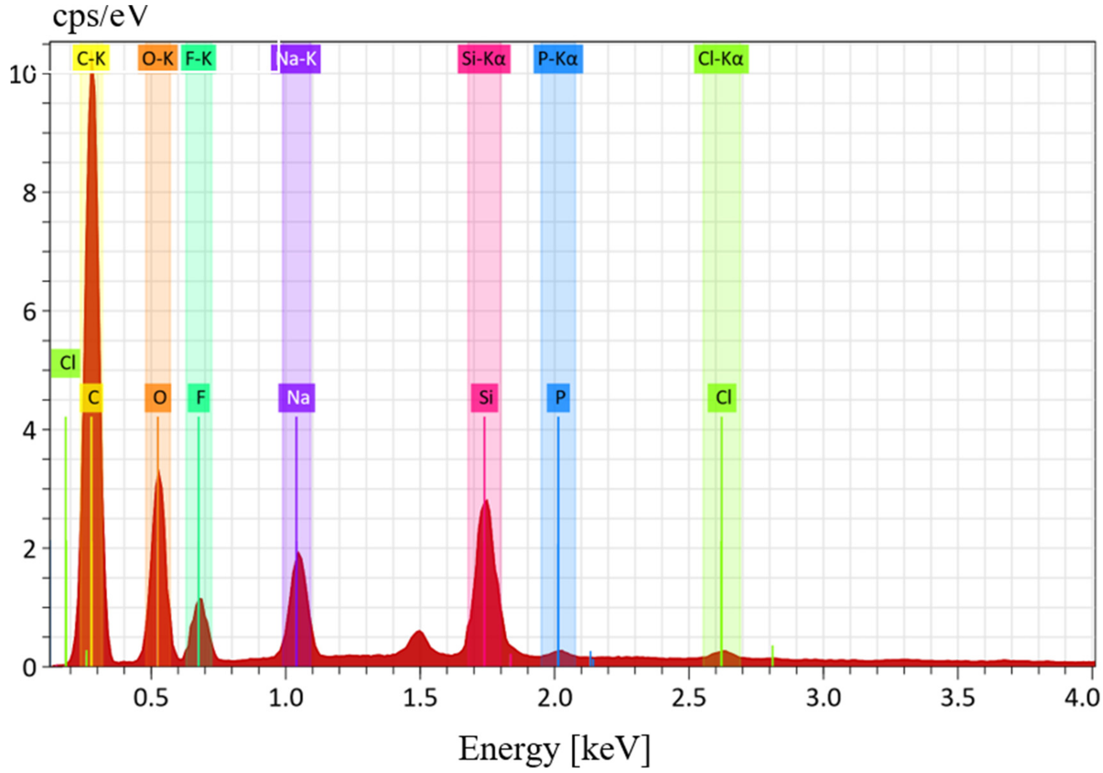

Figure S7. EDX spectrum of the PHB film

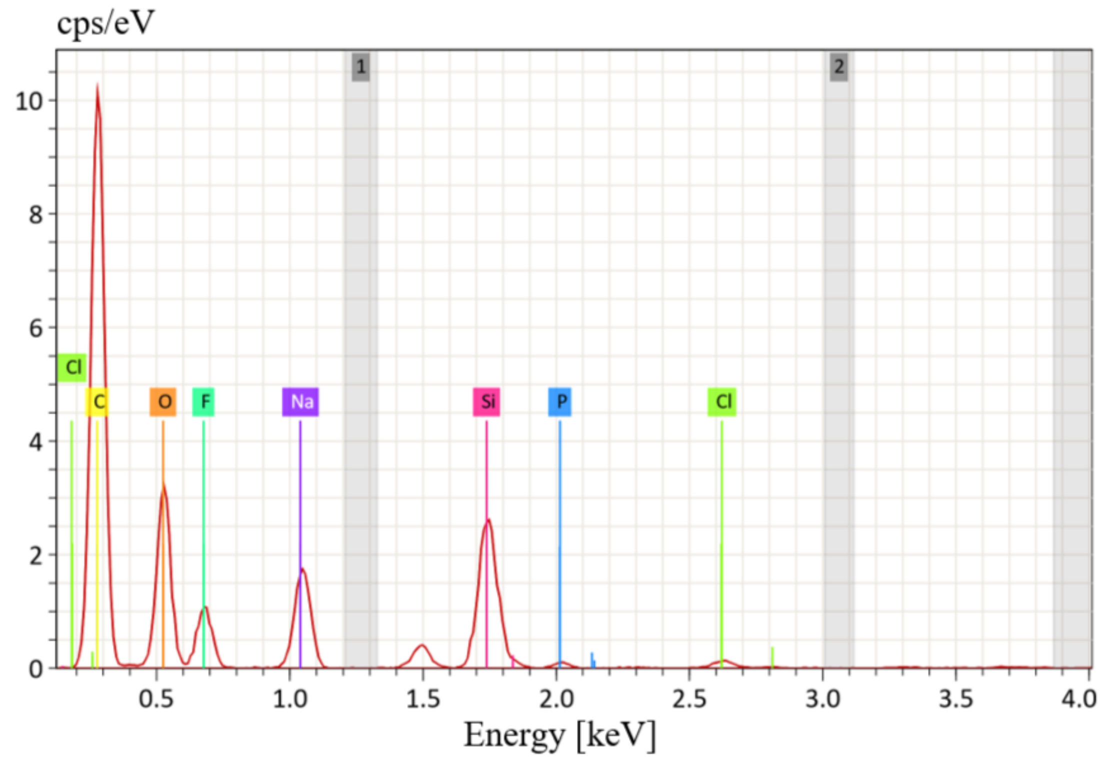

Figure S8. EDX spectrum of the PHB film

Table S4 – Elemental composition of the PHB film

Element	At. No.	Netto	Mass [%]	Mass Norm. [%]	Atom [%]	abs. error [%] (1 sigma)	rel. error [%] (1 sigma)
C	6	151542	57.46	57.46	66.73	6.36	11.07
O	8	51579	26.42	26.42	23.04	3.13	11.86
F	9	18482	7.81	7.81	5.74	1.04	13.26
Na	11	32065	3.71	3.71	2.25	0.26	6.93
Si	14	59304	4.16	4.16	2.07	0.20	4.79
P	15	2115	0.16	0.16	0.07	0.03	20.49
Cl	17	3375	0.29	0.29	0.11	0.04	12.68
		Sum	100.00	100.00	100.00		

8. Energy-dispersive X-ray spectroscopy of *P. yeei* microorganisms coated with a xerogel matrix on a PHB substrate

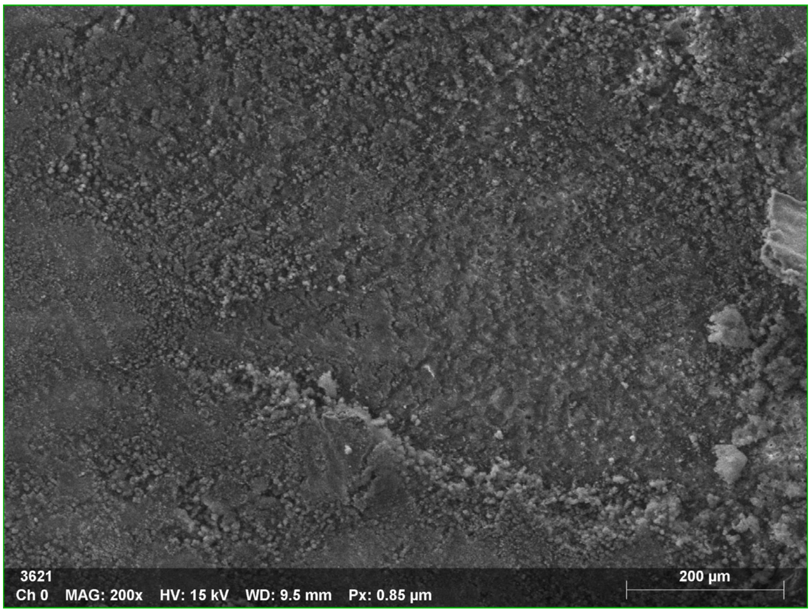

Figure S9. SEM images of *P. yeei* microorganisms coated with a xerogel matrix on a PHB substrate, bar label 200 microns

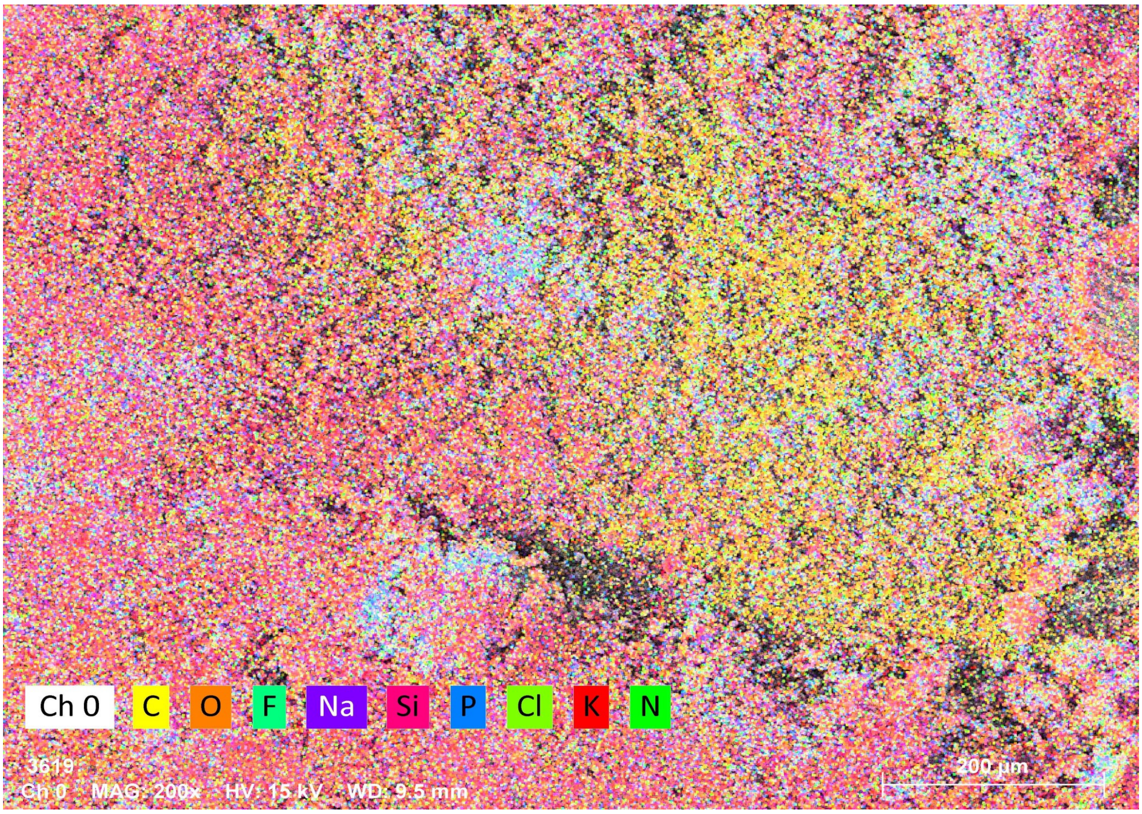

Figure S10. Map of the elements of *P. yeei* microorganisms coated with a xerogel matrix on a PHB substrate, bar label 200 microns

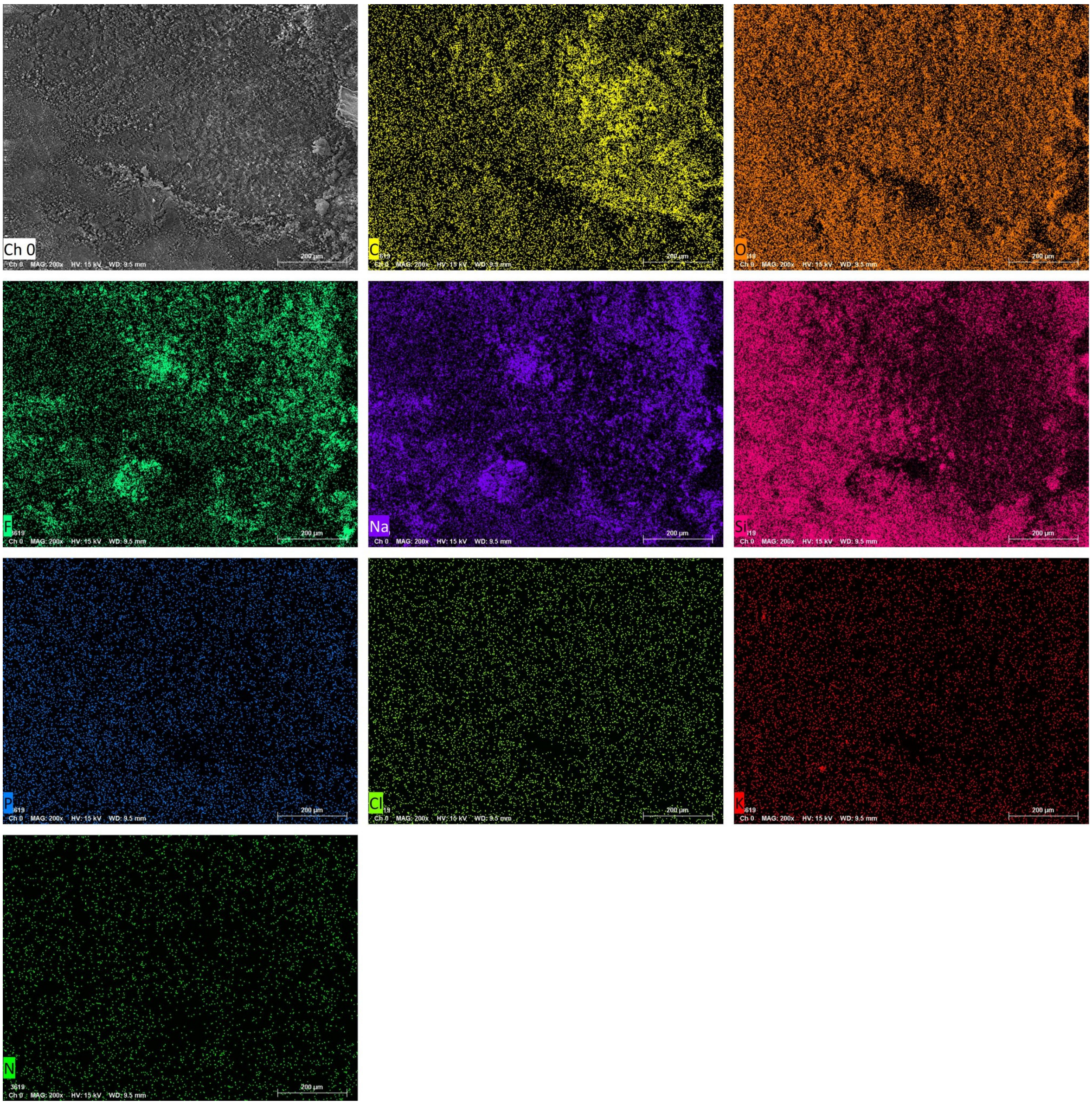

Figure S11. Distribution maps of each of the elements for *P. yeei* microorganisms coated with a xerogel matrix on a PHB substrate, bar label 200 microns

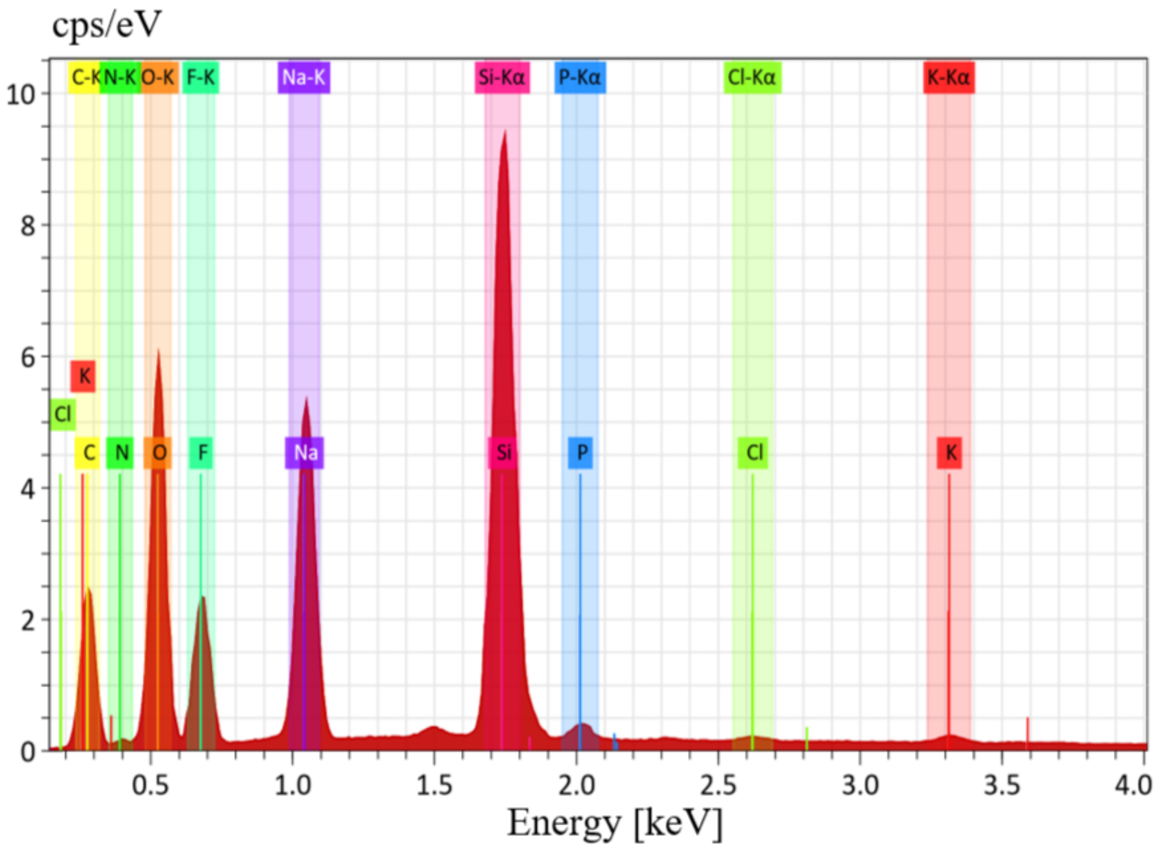

Figure S12. EDX-spectrum of *P. yeei* microorganisms coated with a xerogel matrix on a PHB substrate

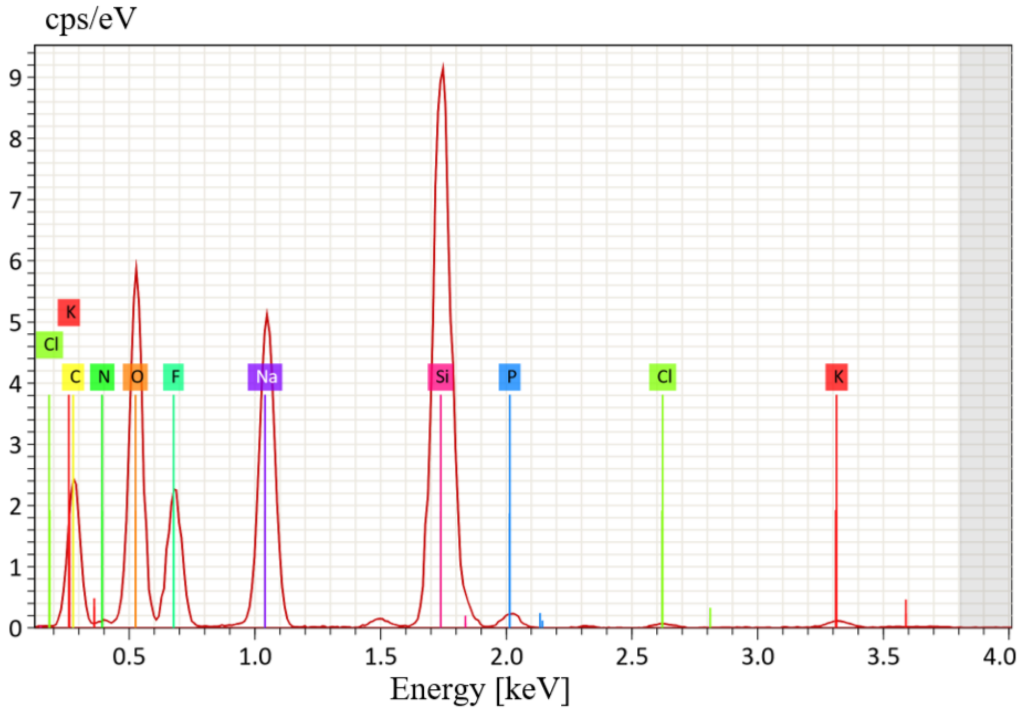

Figure S13. EDX-spectrum of *P. yeei* microorganisms coated with a xerogel matrix on a PHB substrate

Table S5 – Elemental composition of *P. yeei* microorganisms coated with a xerogel matrix on a PHB substrate

Element	At. No.	Netto	Mass [%]	Mass Norm. [%]	Atom [%]	abs. error [%] (1 sigma)	rel. error [%] (1 sigma)
C	6	45970	27.96	25.56	35.30	3.34	11.96
O	8	117320	34.35	31.40	32.56	3.86	11.24
F	9	49088	15.55	14.21	12.41	1.86	11.97
Na	11	118399	12.42	11.35	8.19	0.79	6.40
Si	14	259061	15.73	14.38	8.49	0.68	4.31
P	15	7022	0.49	0.45	0.24	0.05	9.23
Cl	17	2275	0.16	0.15	0.07	0.03	19.39
K	19	3919	0.34	0.31	0.13	0.04	11.07
N	7	2729	2.41	2.20	2.61	0.45	18.69
		Sum	109.41	100.00	100.00		

9. Energy-dispersive X-ray spectroscopy of *P. yeei* microorganisms coated with a xerogel matrix extracted from a PHB substrate

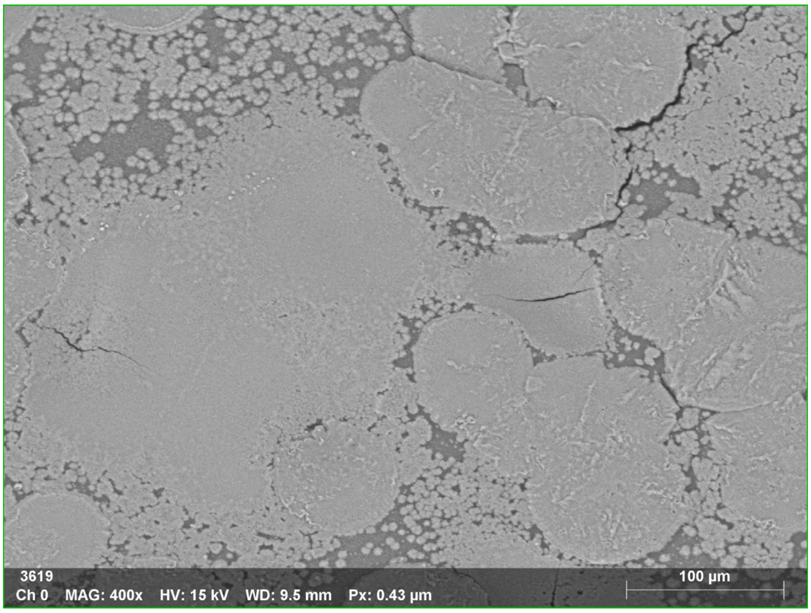

Figure S14. SEM-image of *P. yeei* microorganisms coated with a xerogel matrix extracted from a PHB substrate

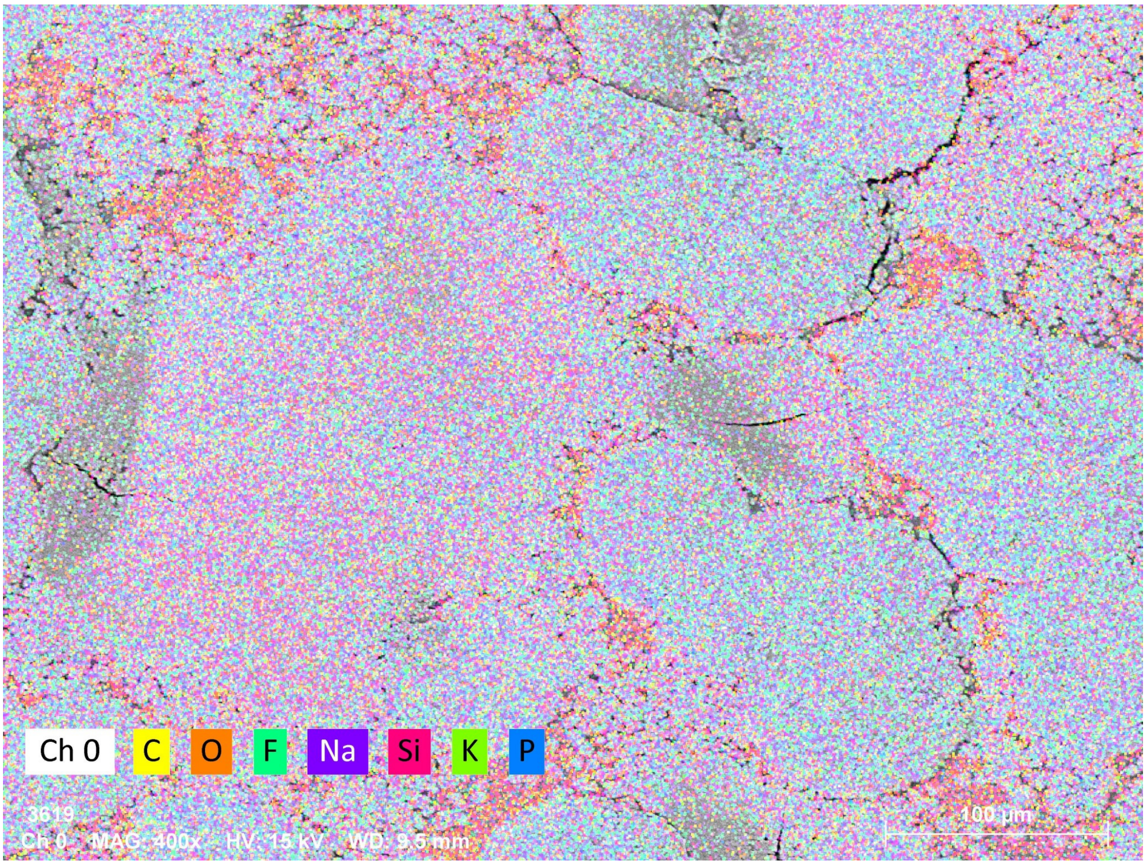

Figure S15. An element map of *P. yeei* microorganisms coated with a xerogel matrix extracted from a PHB substrate

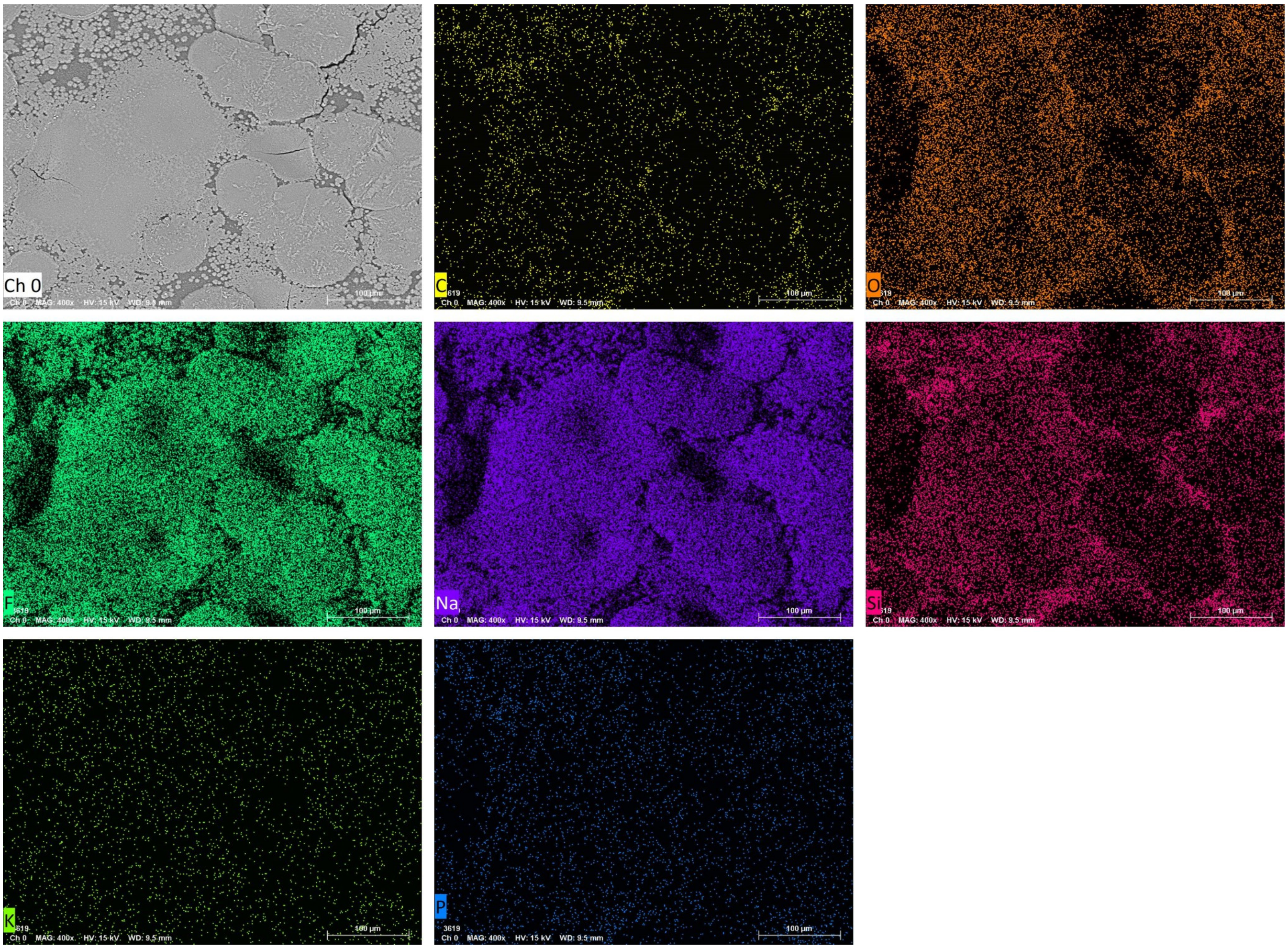

Figure S16. Distribution maps of each of the elements for *P. yeei* microorganisms coated with a xerogel matrix extracted from a PHB substrate

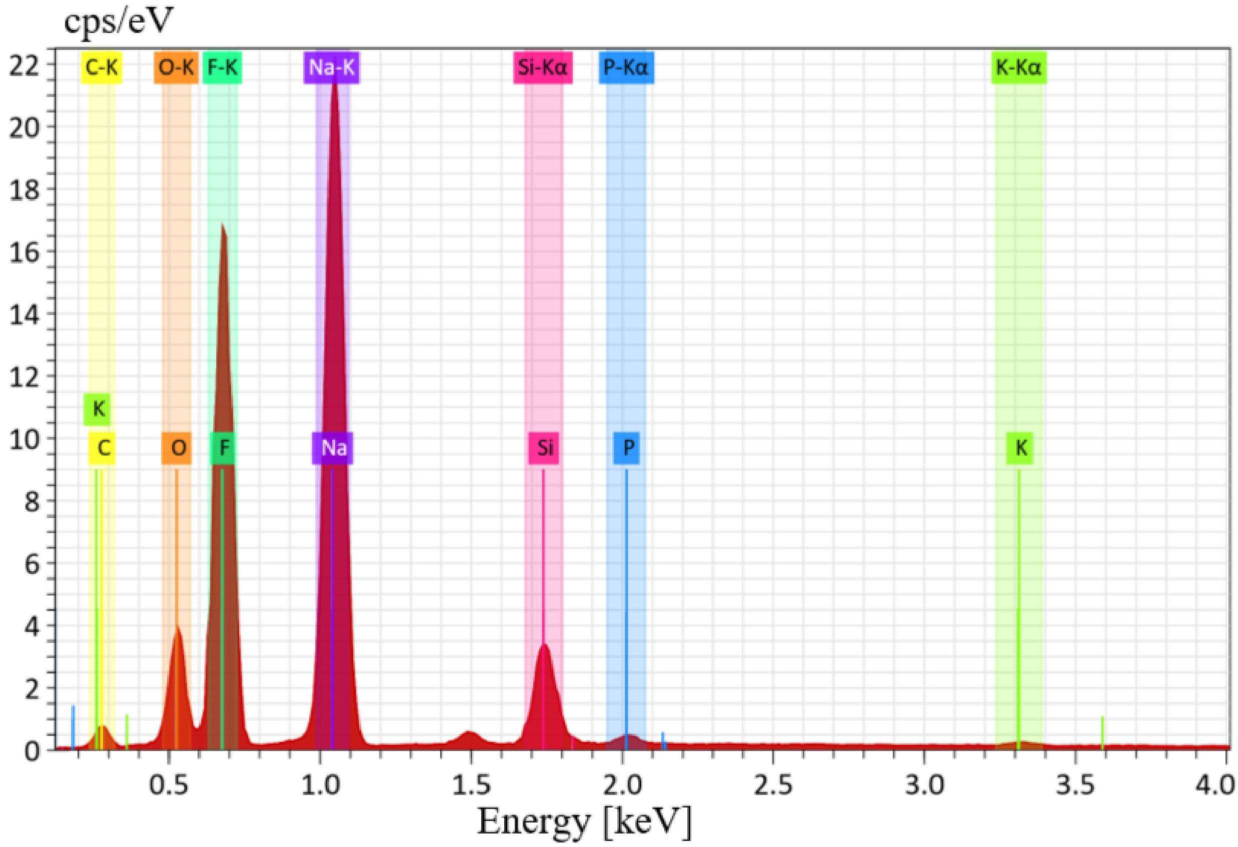

Figure S17. EDX-spectrum of *P. yeei* microorganisms coated with a xerogel matrix extracted from a PHB substrate

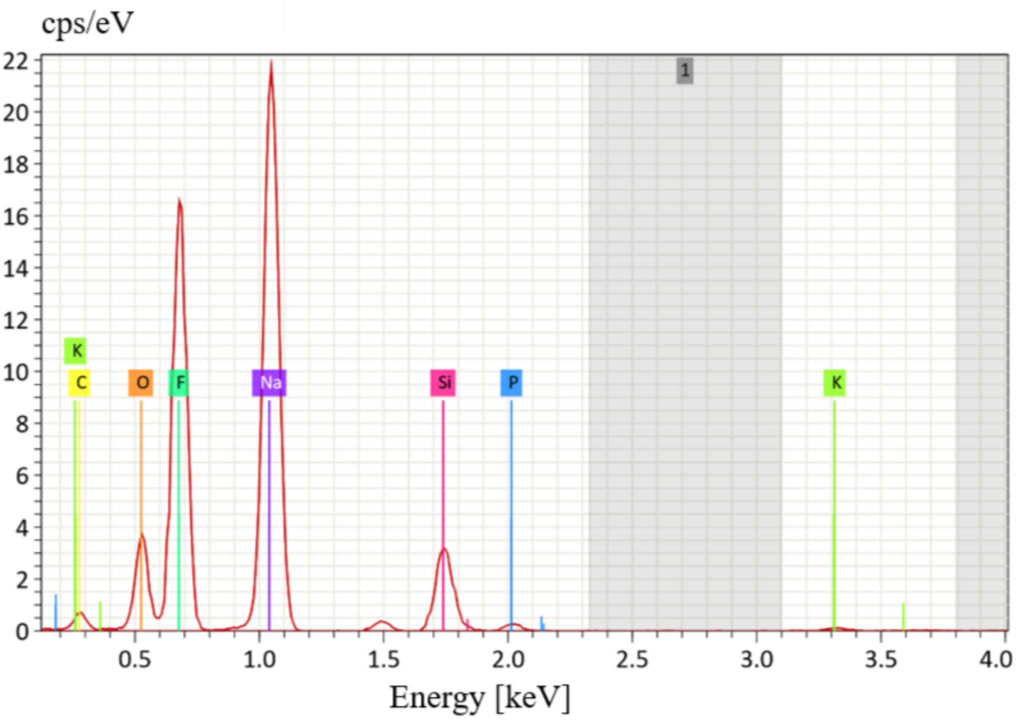

Figure S18. EDX-spectrum of *P. yeei* microorganisms coated with a xerogel matrix extracted from a PHB substrate

Table S6 – Elemental composition of *P. yeei* microorganisms coated with a xerogel matrix extracted from a PHB substrate

Element	At. No.	Netto	Mass [%]	Mass Norm. [%]	Atom [%]	abs. error [%] (1 sigma)	rel. error [%] (1 sigma)
C	6	4161	6.98	6.35	10.37	1.16	16.56
O	8	25928	10.89	9.90	12.14	1.38	12.71
F	9	121805	42.74	38.84	40.12	4.79	11.20
Na	11	171589	43.00	39.08	33.37	2.69	6.25
Si	14	31260	5.63	5.12	3.58	0.26	4.67
K	19	1307	0.27	0.25	0.13	0.04	13.58
P	15	2897	0.52	0.47	0.30	0.05	9.36
		Sum	110.04	100.00	100.00		

10. Scanning electron microscopy of a PHB film with *P.yeei* microorganisms adsorbed on it, coated with a xerogel matrix

This section contains additional SEM images that provide further evidence for the formation and homogeneity of the PHB-xerogel biocomposite, supporting the morphological analysis discussed in Section 2.1 of the main manuscript.

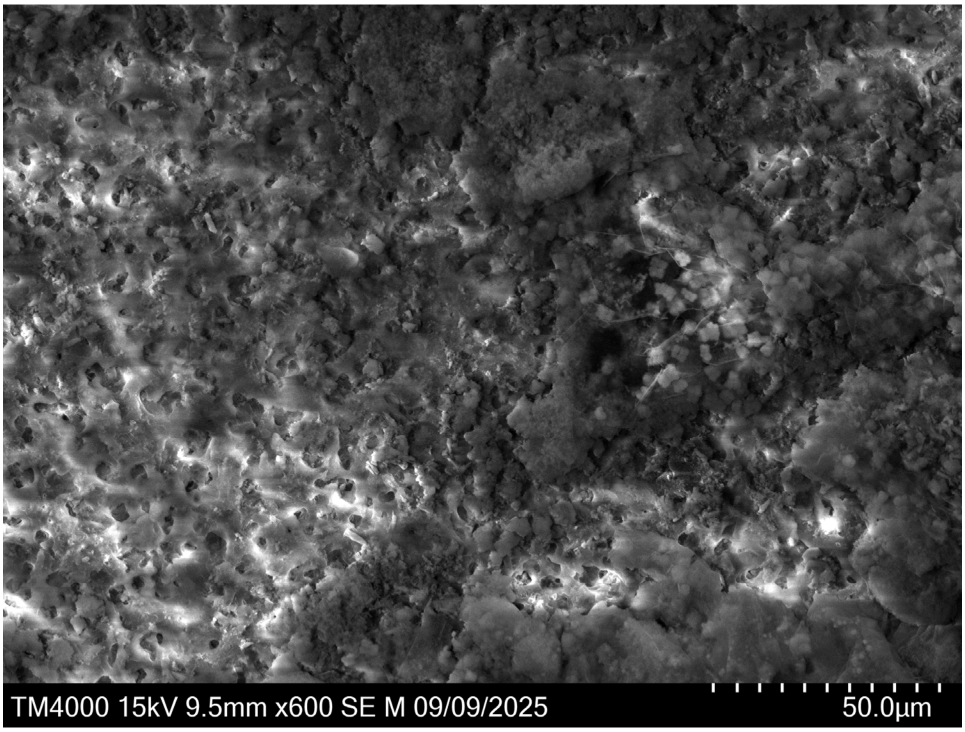

Figure S19. SEM-image of a PHB film with *P.yeei* microorganisms adsorbed on it, coated with a xerogel matrix, 50 microns bar label (SE mode)

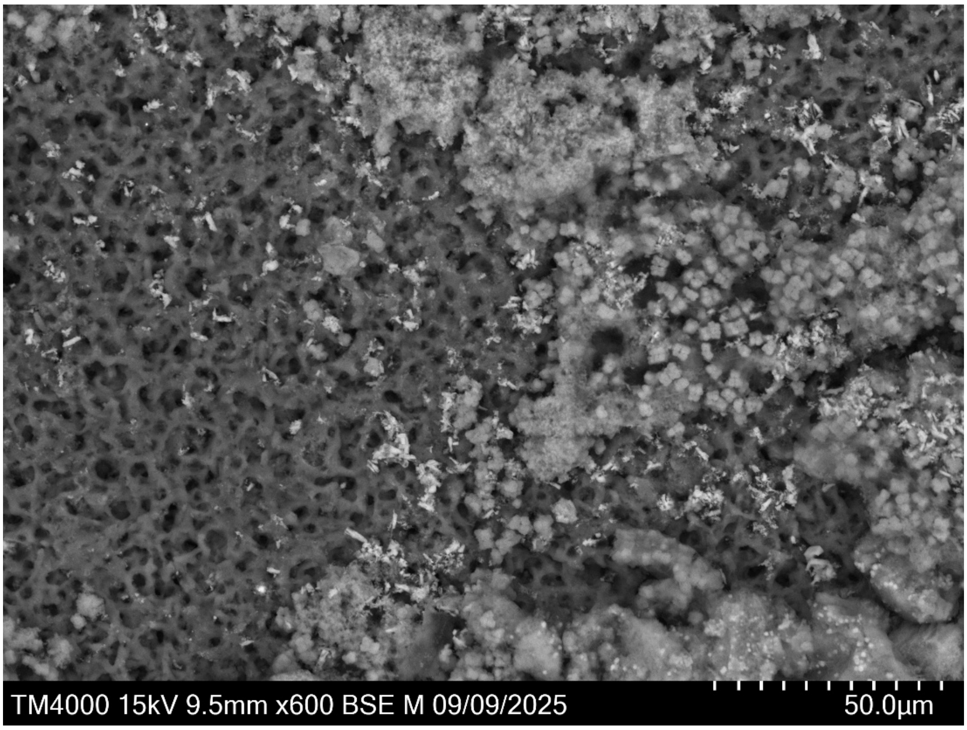

Figure S20. SEM-image of a PHB film with *P.yeei* microorganisms adsorbed on it, coated with a xerogel matrix, 50 microns bar label (BSE mode)

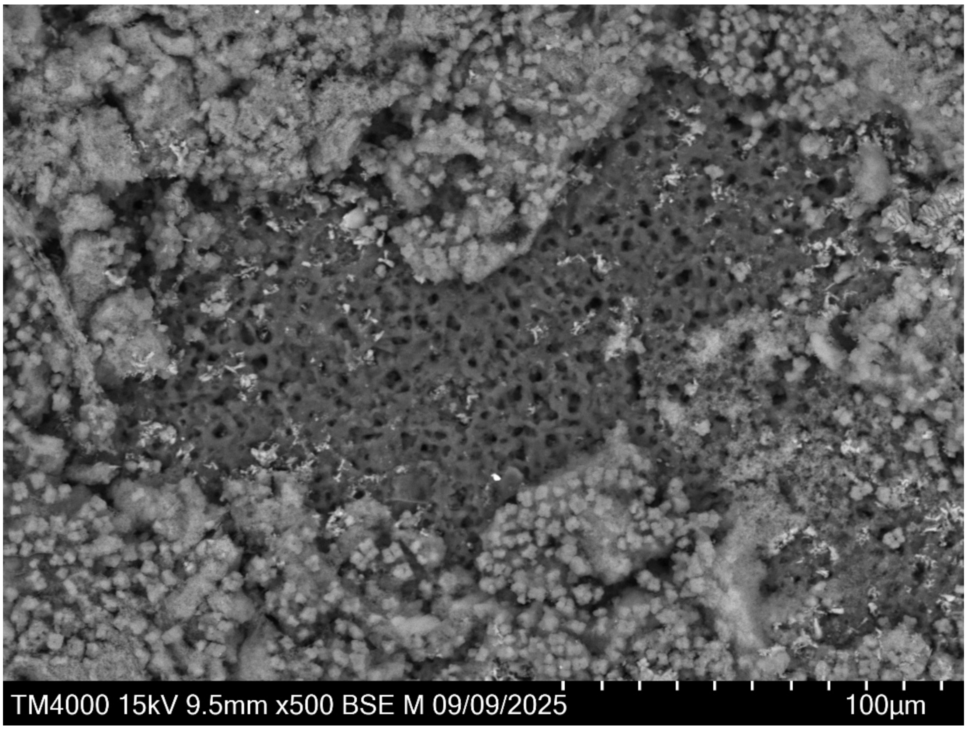

Figure S21. SEM-image of a PHB film with *P.yeei* microorganisms adsorbed on it, coated with a xerogel matrix, bar label 100 microns (SE mode)

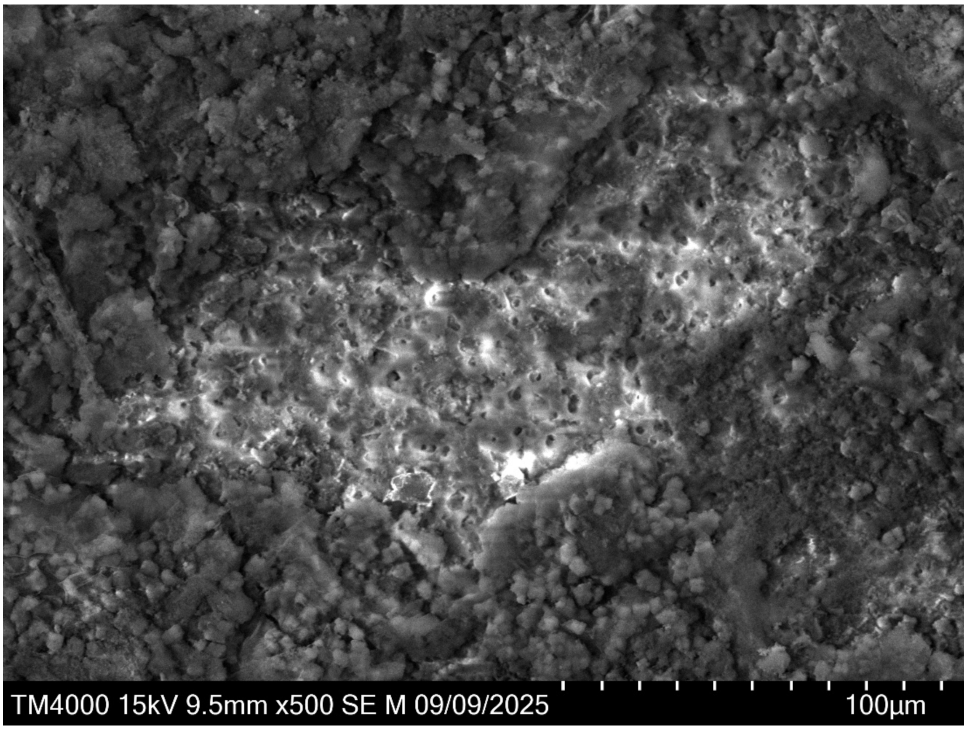

Figure S22. SEM-image of a PHB film with *P.yeei* microorganisms adsorbed on it, coated with a xerogel matrix, bar label 100 microns (BSE mode)

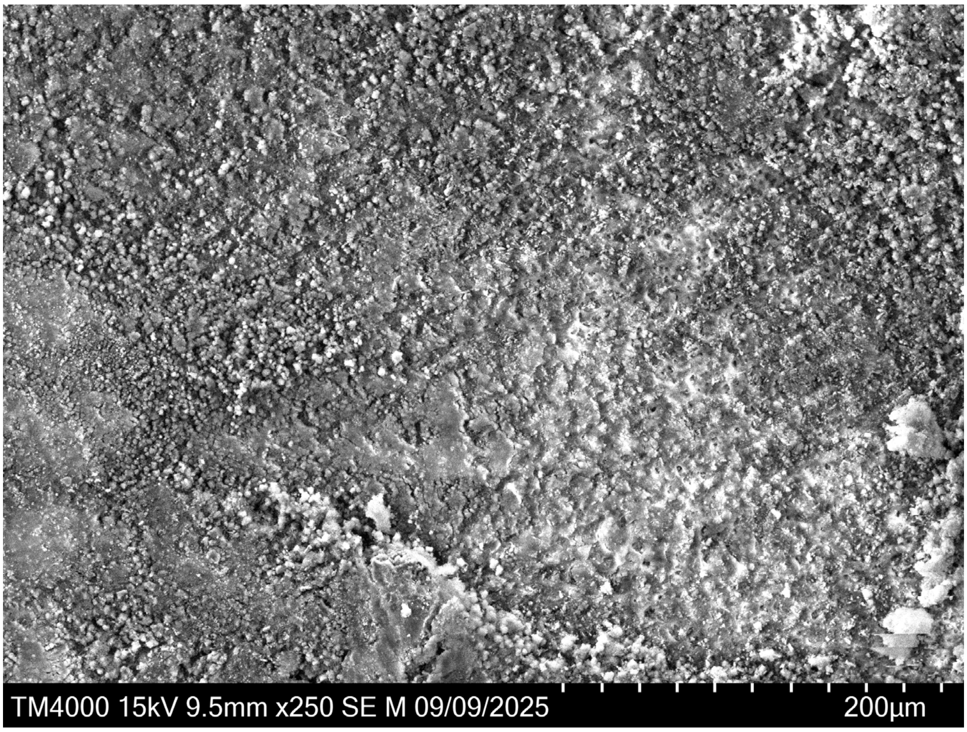

Figure S23. SEM-image of a PHB film with *P.yeei* microorganisms adsorbed on it, coated with a xerogel matrix, 200 microns bar label (SE mode)

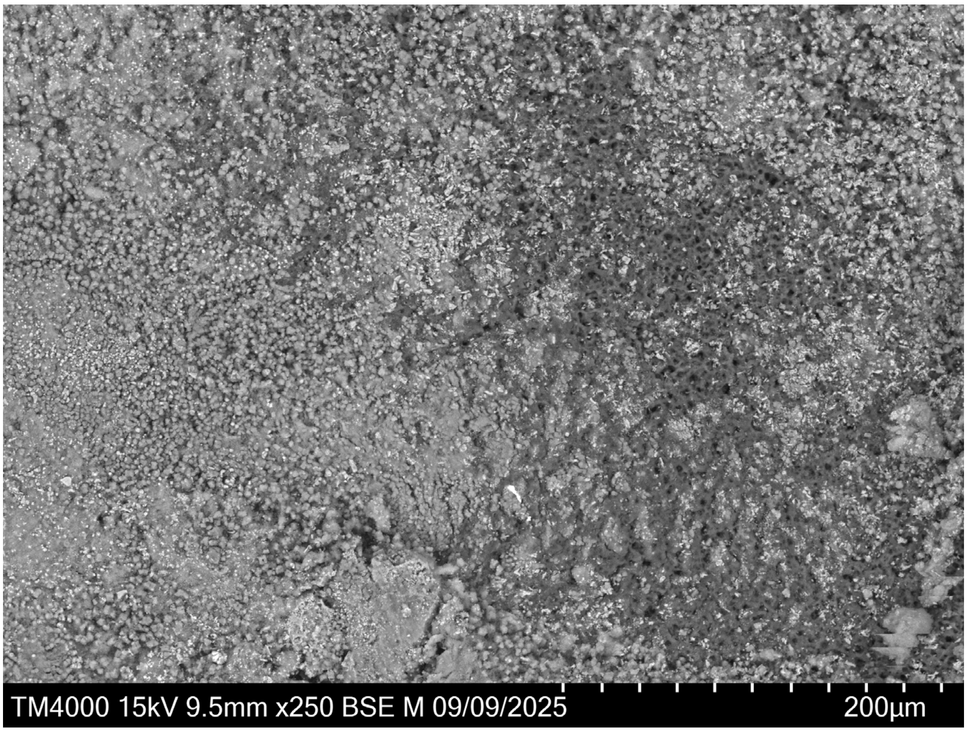

Figure S24. SEM-image of a PHB film with *P.yeei* microorganisms adsorbed on it, coated with a xerogel matrix, bar label 200 microns (BSE mode)

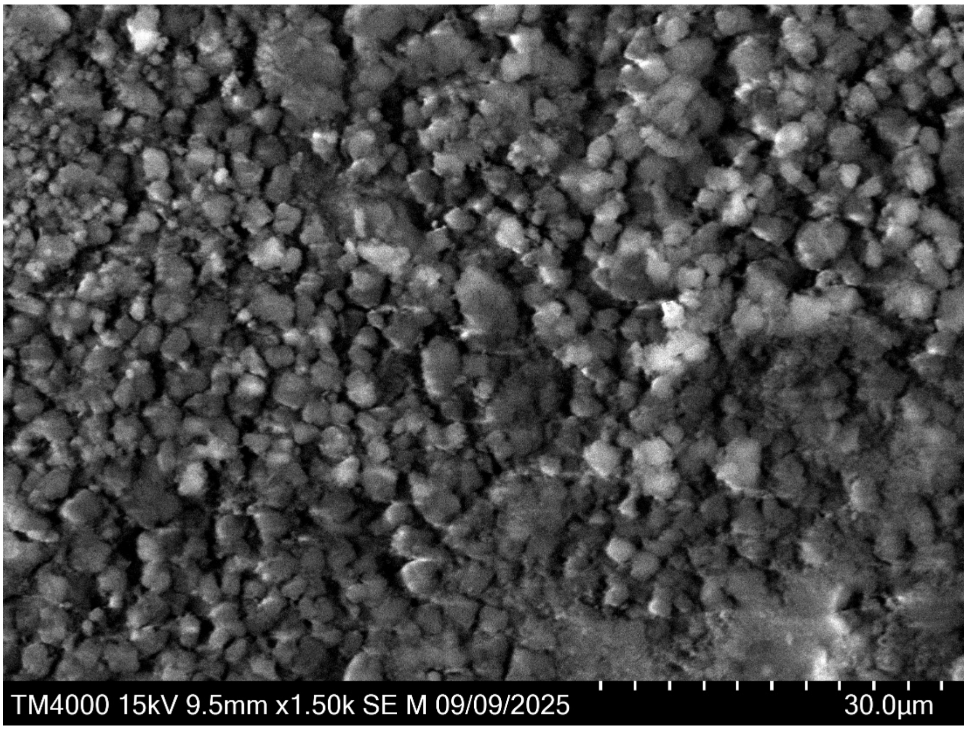

Figure S25. SEM-image of a PHB film with *P.yeei* microorganisms adsorbed on it, coated with a xerogel matrix, 30 microns bar label (SE mode)

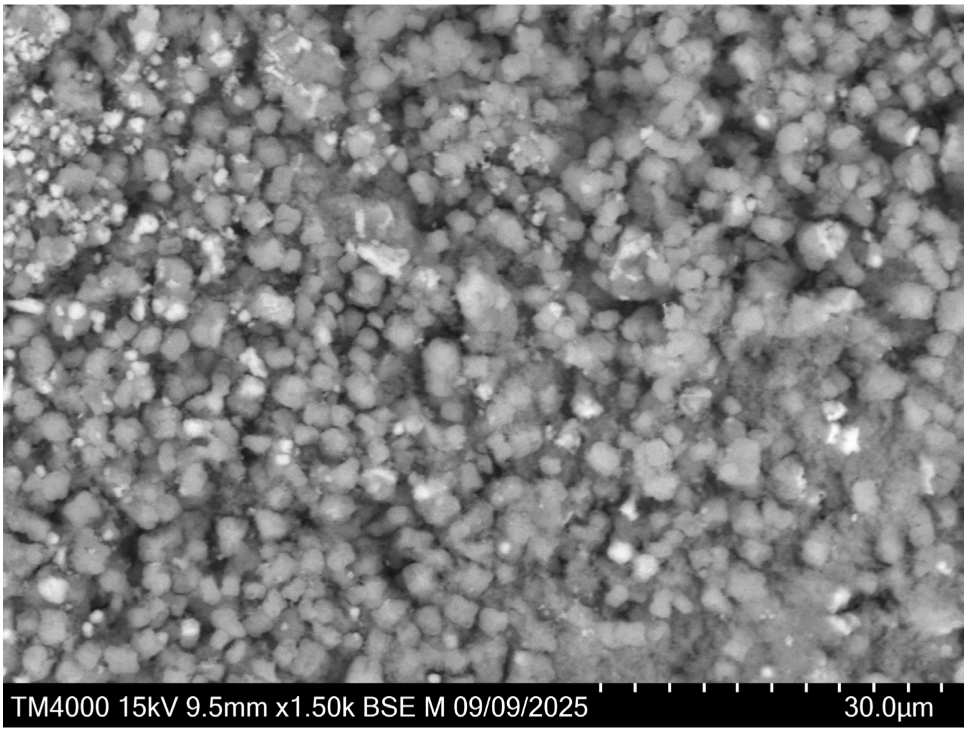

Figure S26. SEM-image of a PHB film with *P.yeei* microorganisms adsorbed on it, coated with a xerogel matrix, bar label 30 microns (BSE mode)

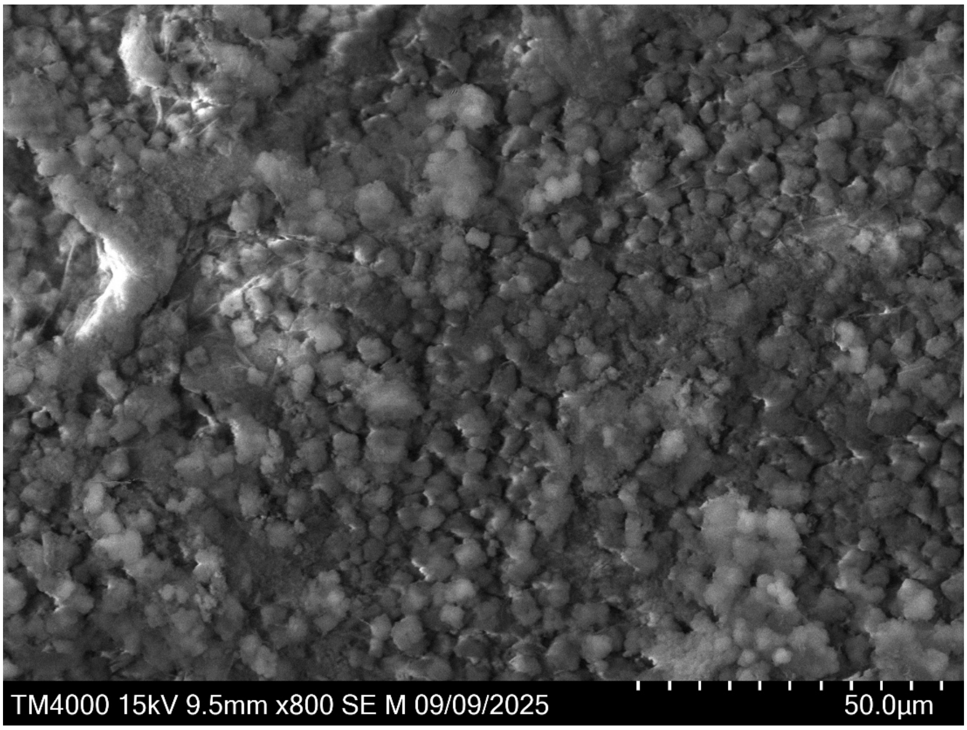

Figure S27. SEM-image of a PHB film with *P.yeei* microorganisms adsorbed on it, coated with a xerogel matrix, 50 microns bar label (SE mode)

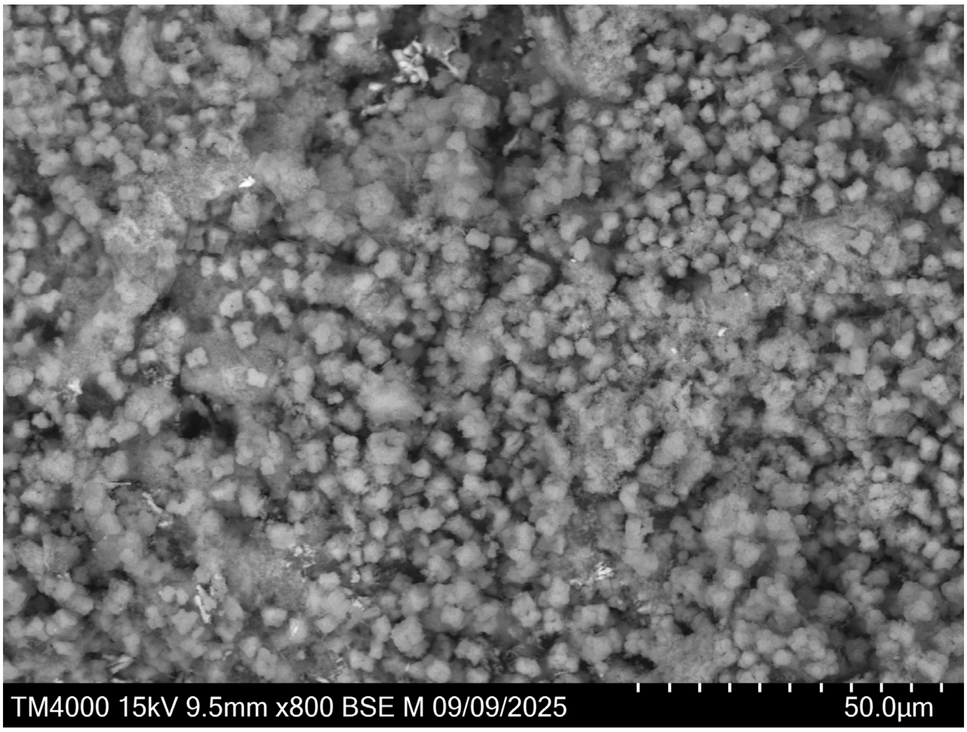

Figure S28. SEM-image of a PHB film with *P.yeei* microorganisms adsorbed on it, coated with a xerogel matrix, 50 microns bar label (BSE mode)

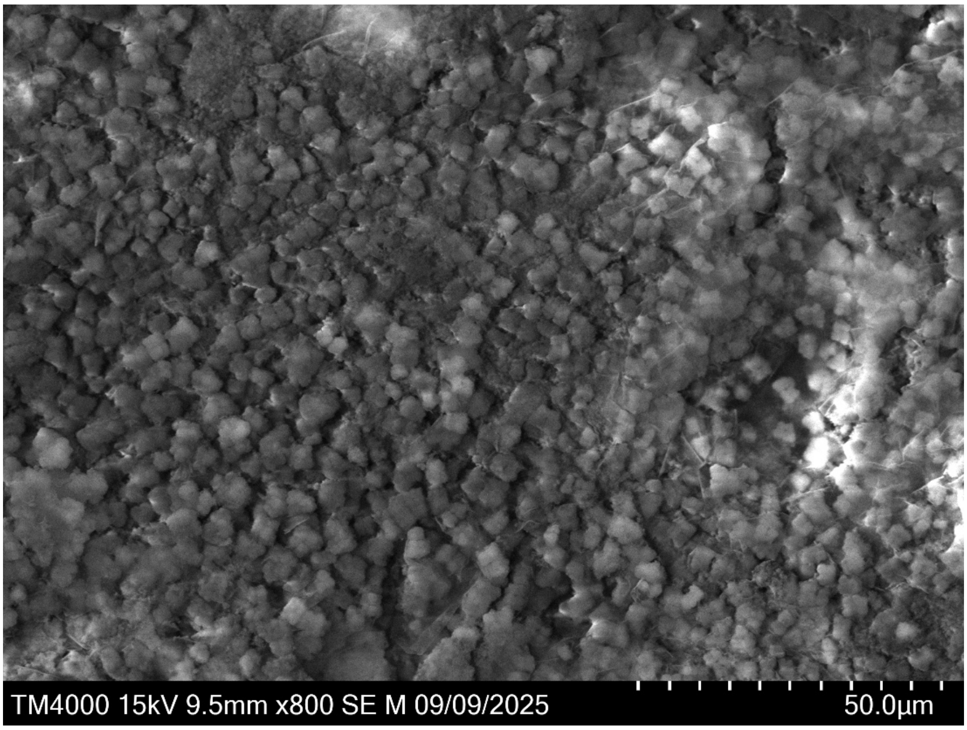

Figure S29. SEM-image of a PHB film with *P.yeei* microorganisms adsorbed on it, coated with a xerogel matrix, 50 microns bar label (SE mode)

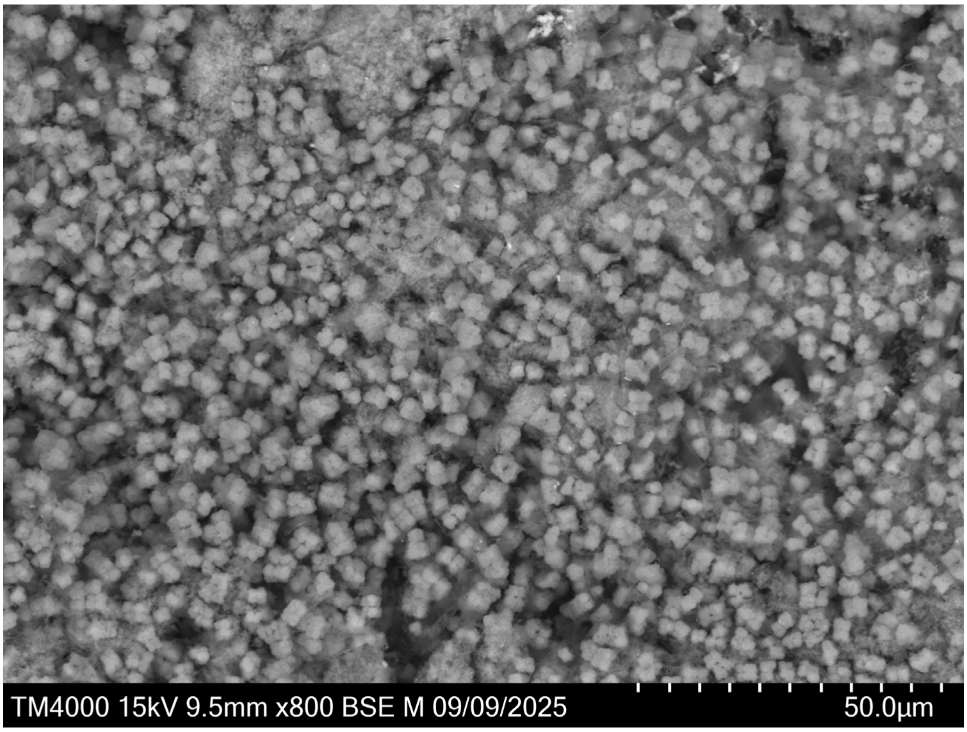

Figure S30. SEM-image of a PHB film with *P.yeei* microorganisms adsorbed on it, coated with a xerogel matrix, 50 microns bar label (BSE mode)

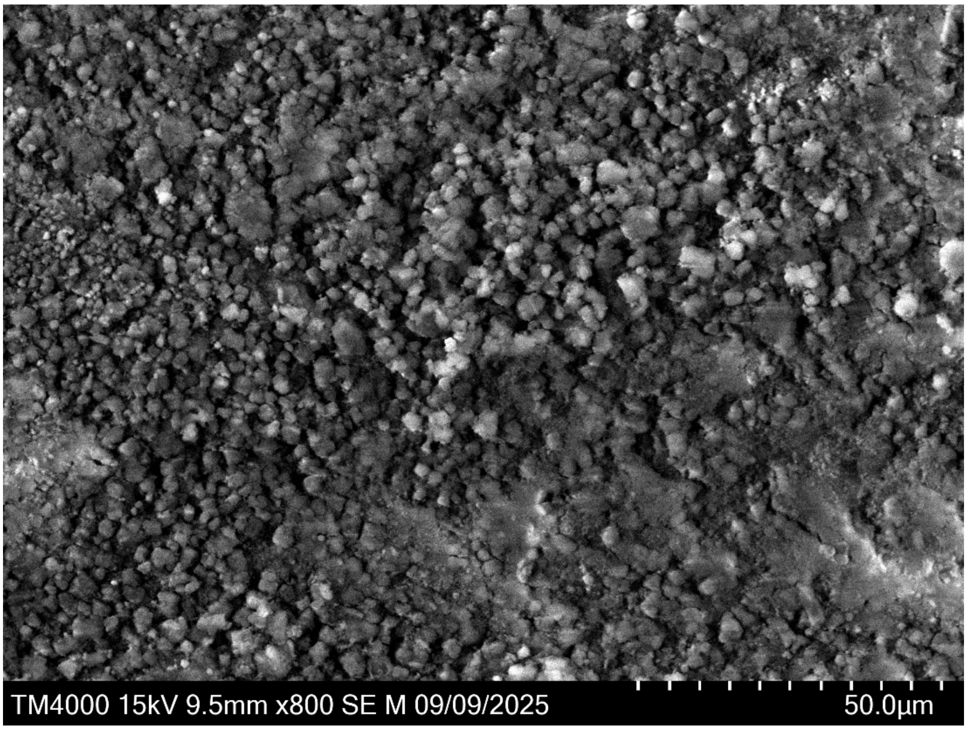

Figure S31. SEM-image of a PHB film with *P.yeei* microorganisms adsorbed on it, coated with a xerogel matrix, 50 microns bar label (SE mode)

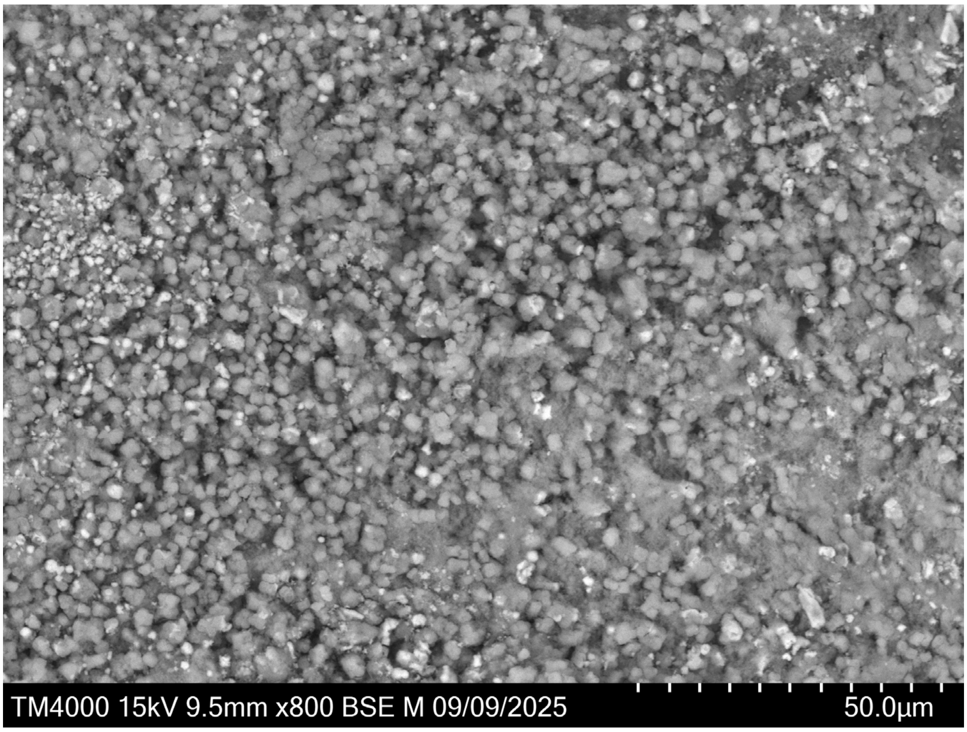

Figure S32. SEM-image of a PHB film with *P.yeei* microorganisms adsorbed on it, coated with a xerogel matrix, 50 microns bar label (BSE mode)

11. Scanning electron microscopy of a dried bioreceptor element formed in the work

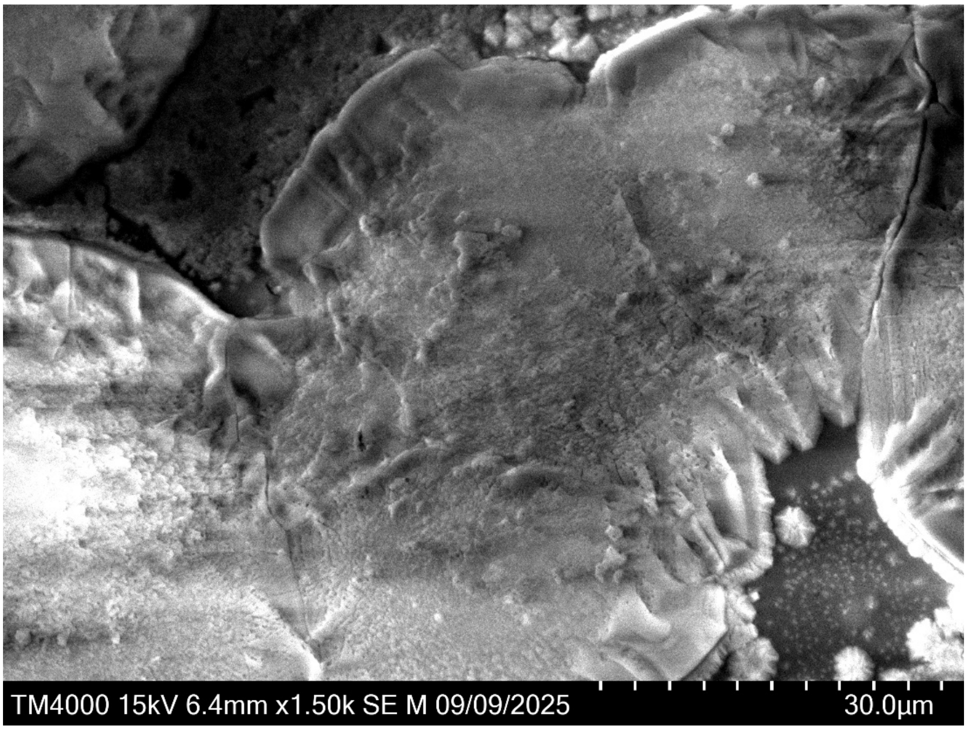

Figure S33. SEM is an image of a dried bioreceptor element formed in operation, bar label 30 microns (SE mode)

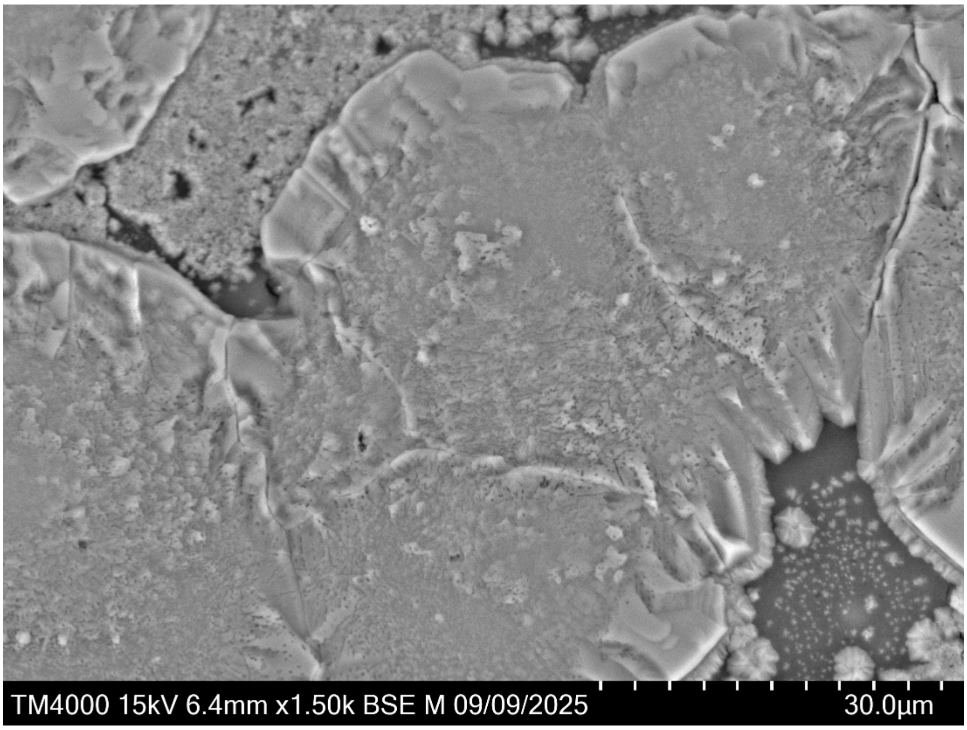

Figure S34. SEM-image of a dried bioreceptor element formed in operation, bar label 30 microns (BSE mode)

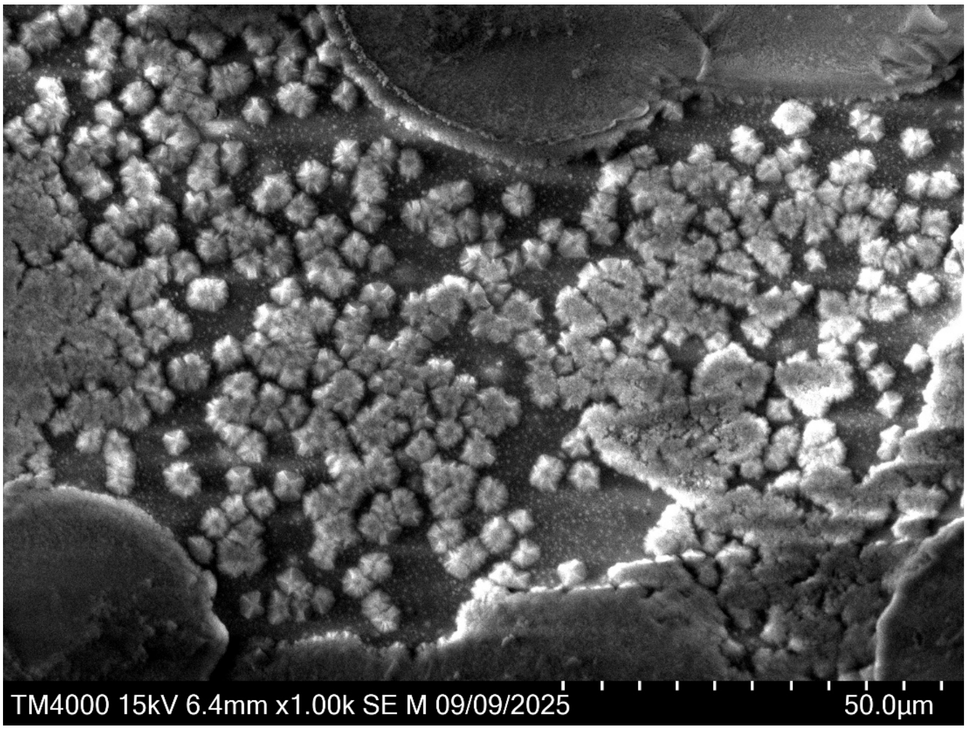

Figure S35. SEM-image of a dried bioreceptor element formed in operation, 50 microns bar (SE mode)

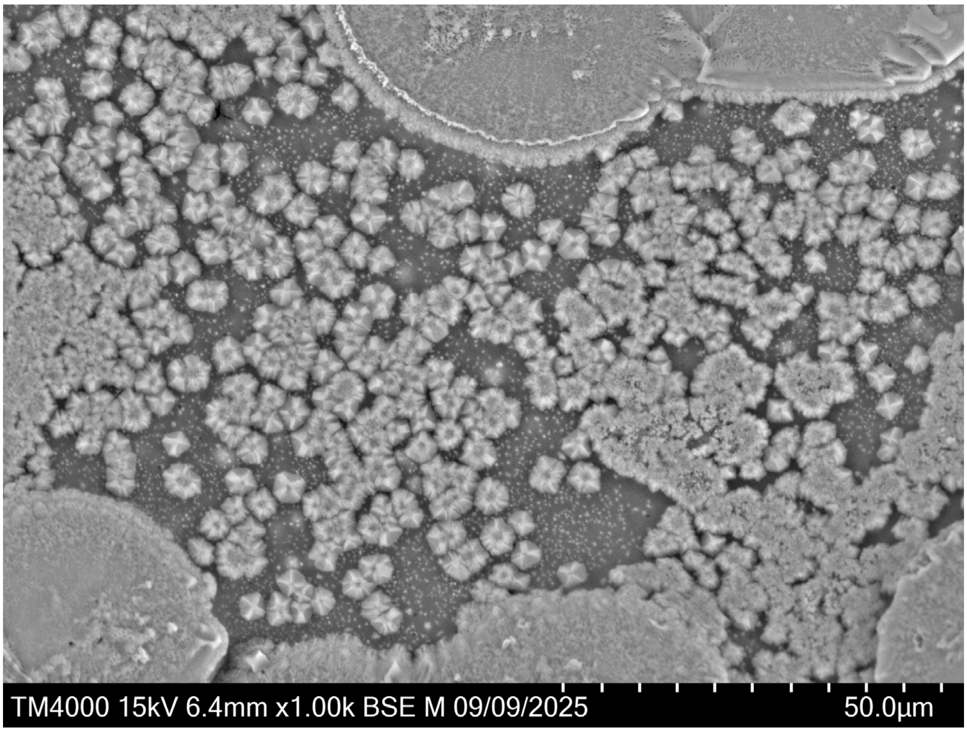

Figure S36. SEM-image of a dried bioreceptor element formed in operation, 50 microns bar label (BSE mode)

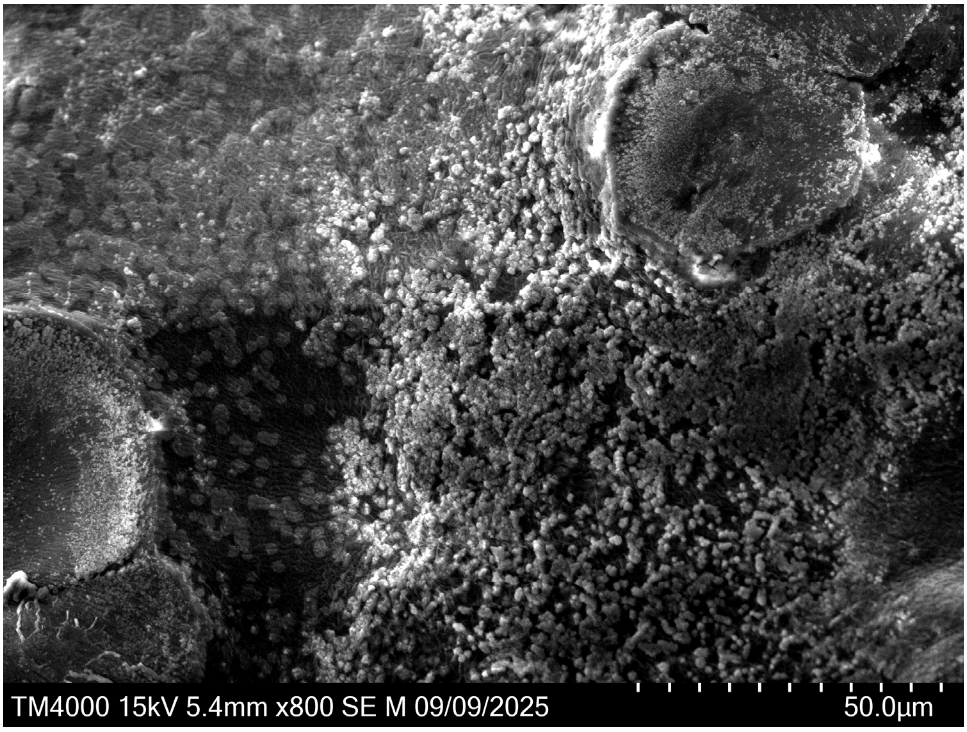

Figure S37. SEM-image of a dried bioreceptor element formed in operation, 50 microns bar mark (SE mode)

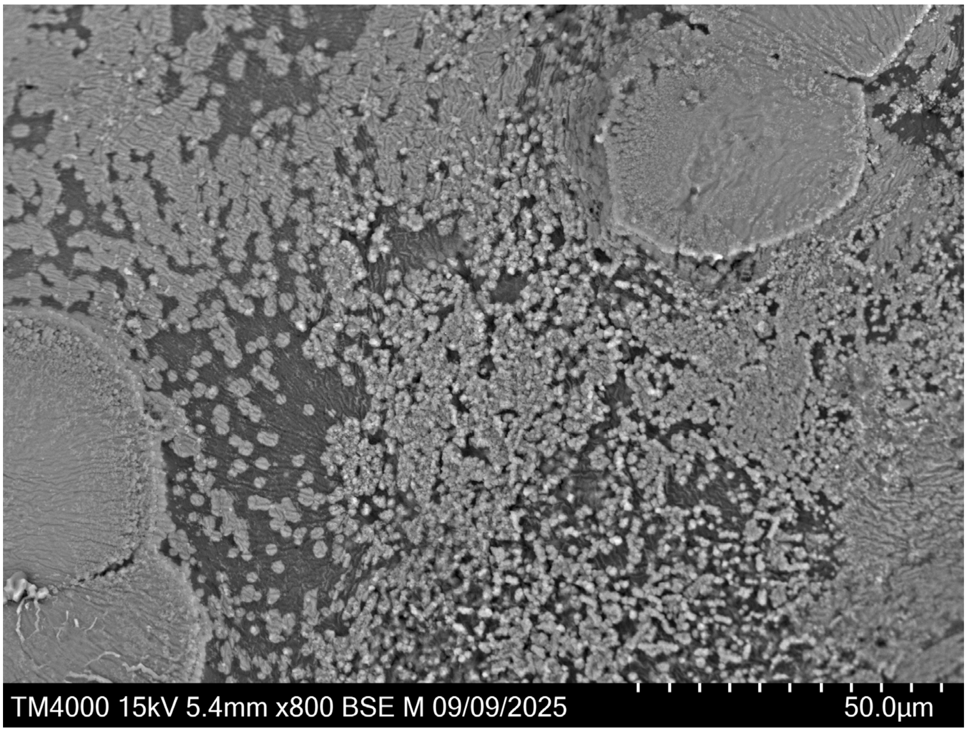

Figure S38. SEM-image of a dried bioreceptor element formed in operation, 50 microns bar (BSE mode)

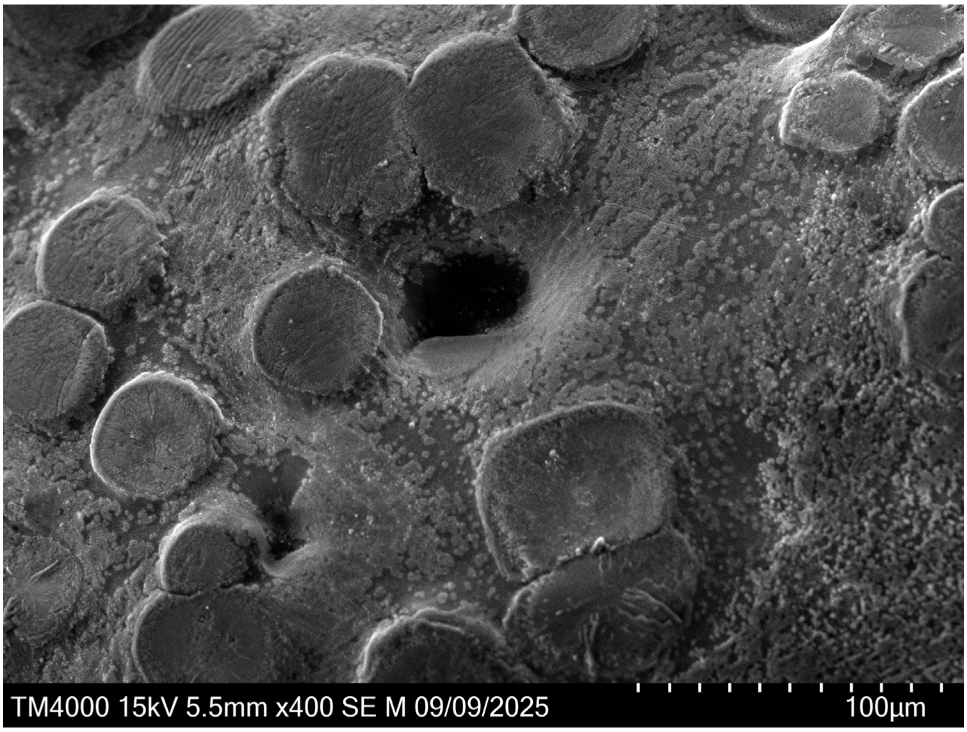

Figure S39. SEM is an image of a dried bioreceptor element formed in operation, bar label 100 microns (SE mode)

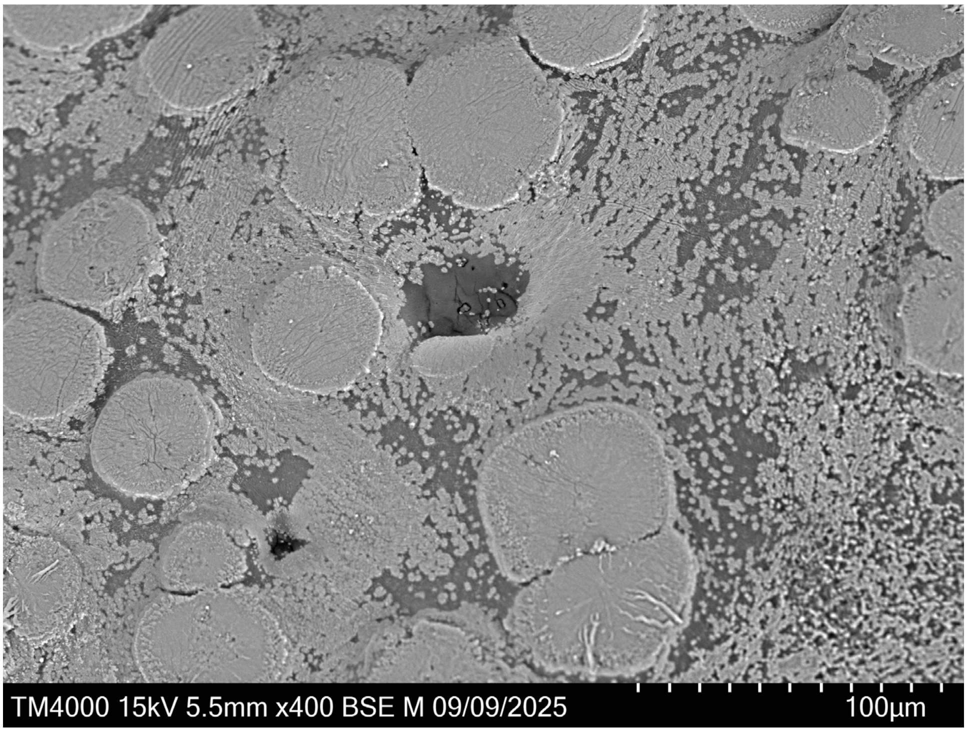

Figure S40. SEM-image of a dried bioreceptor element formed in operation, bar label 100 microns (BSE mode)

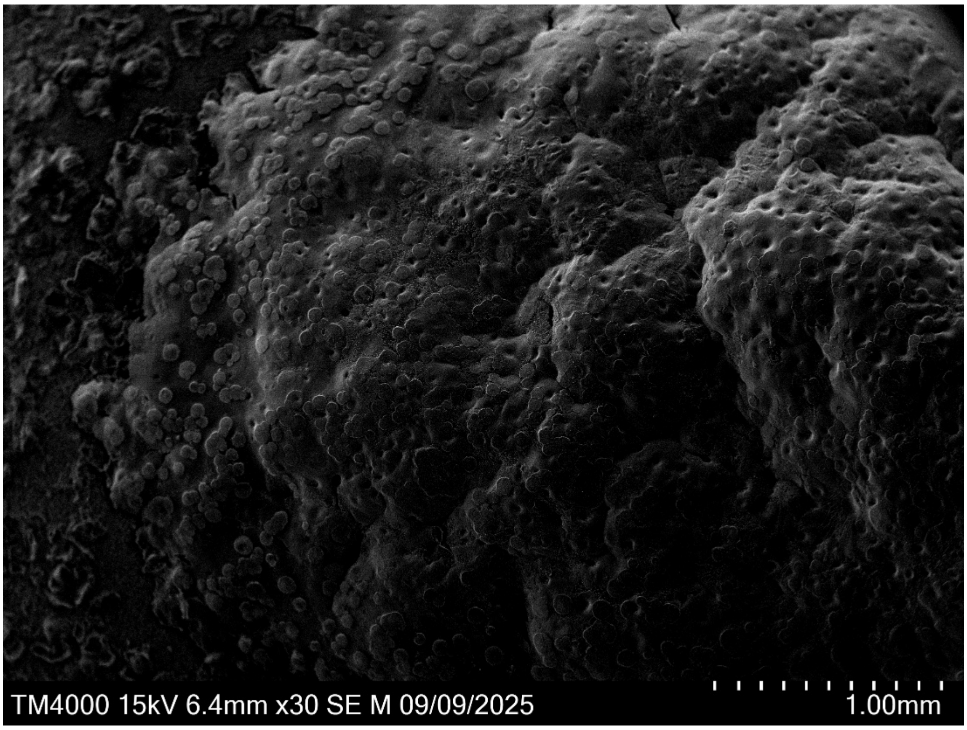

Figure S41. SEM-image of a dried bioreceptor element formed in operation, 1 mm bar label (SE mode)

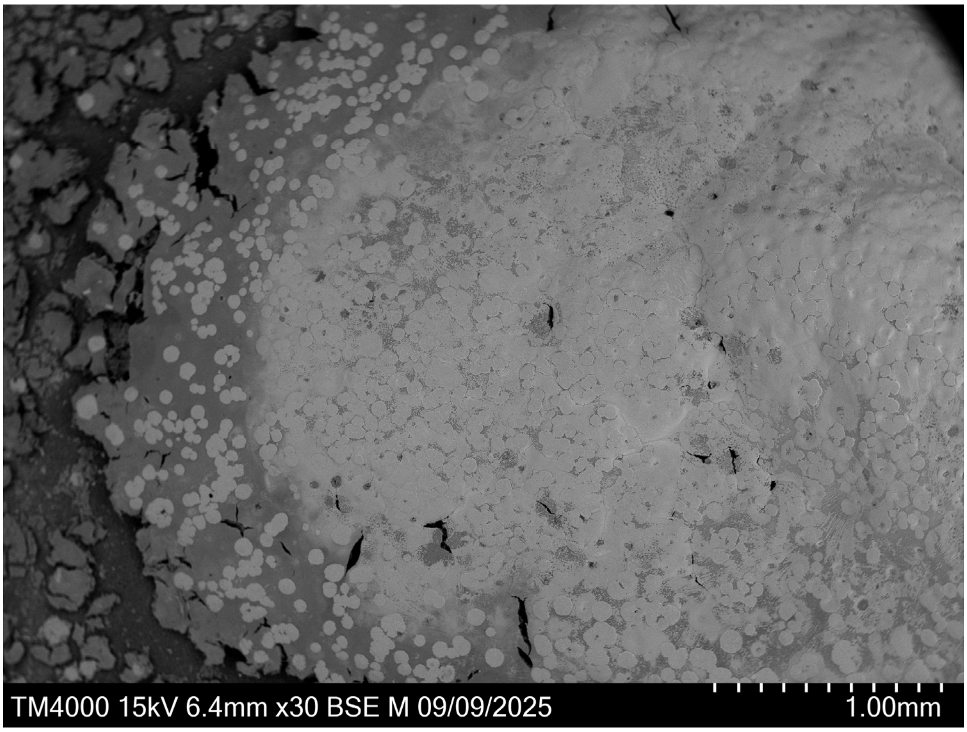

Figure S42. SEM-image of a dried bioreceptor element formed in operation, 1 mm bar label (BSE mode)

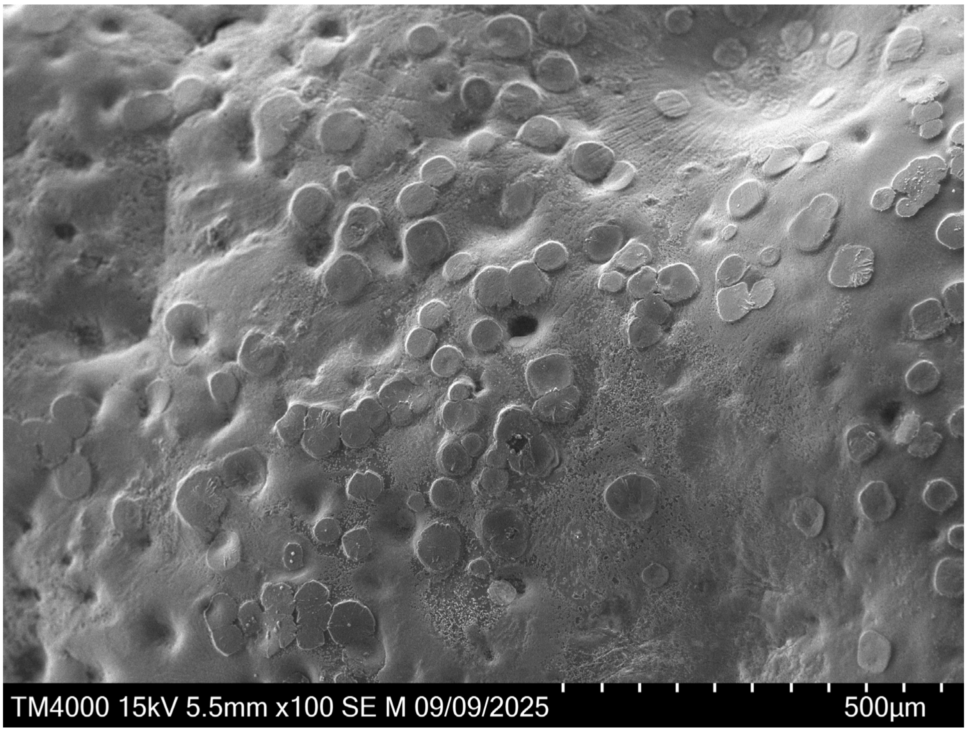

Figure S43. SEM-image of a dried bioreceptor element formed in operation, 500 microns bar (SE mode)

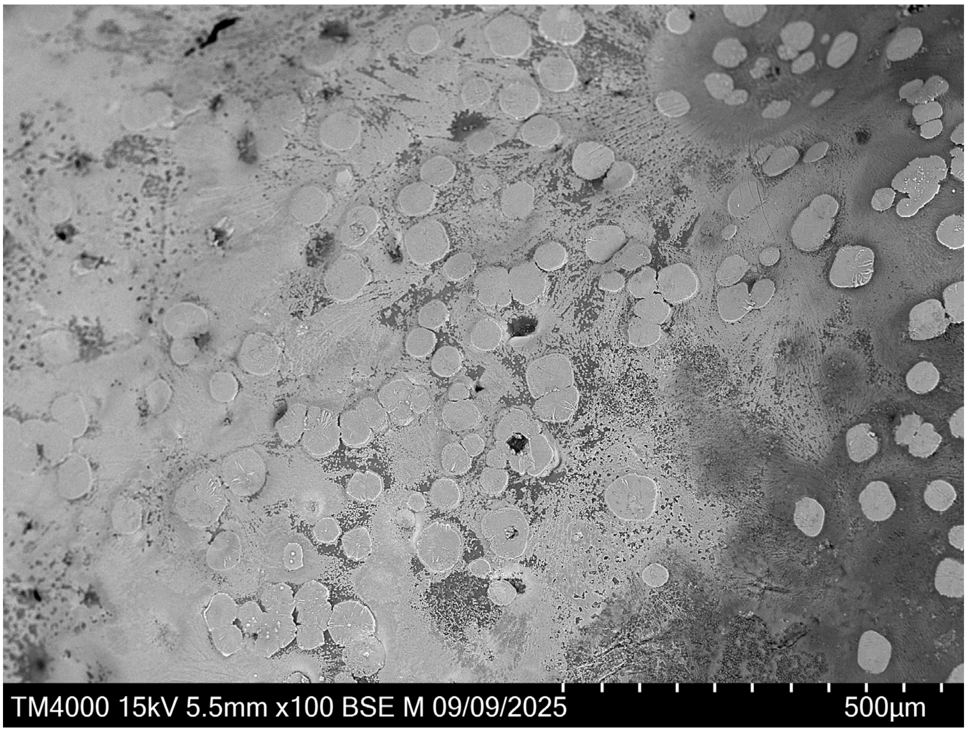

Figure S44. SEM is an image of a dried bioreceptor element formed in operation, with a 500 micron bar (Section 6; BSE mode)

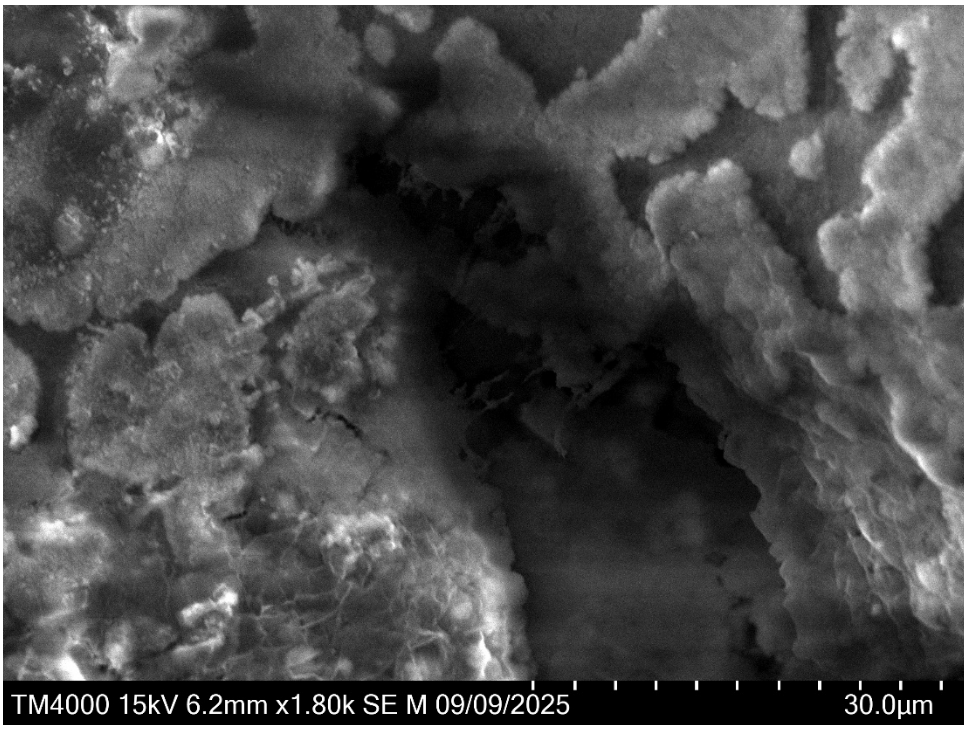

Figure S45. SEM is an image of a dried bioreceptor element formed in operation, bar label 30 microns (SE mode)

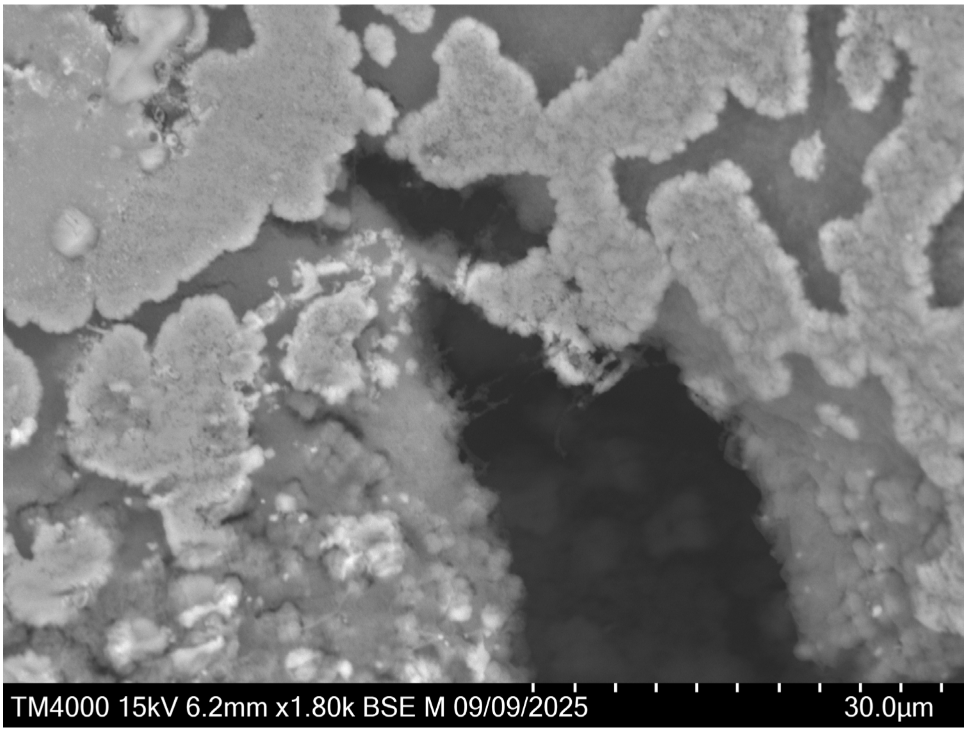

Figure S46. SEM is an image of a dried bioreceptor element formed in operation, a 30 micron bar mark (BSE mode)

12. Long-term stability of the biosensor

The long-term operational stability was quantitatively assessed over 22 days (Figure S47). The biosensor maintained 78 % of its initial response after 7 days, 76% after 14 days, and 64% after 20 days of regular operation. The response decreased to 47% on day 21. Following the established criterion of 50% response retention for determining operational lifespan, the biosensor demonstrated a service life of 20 days, corresponding to approximately 80-100 measurement cycles under standard operating conditions

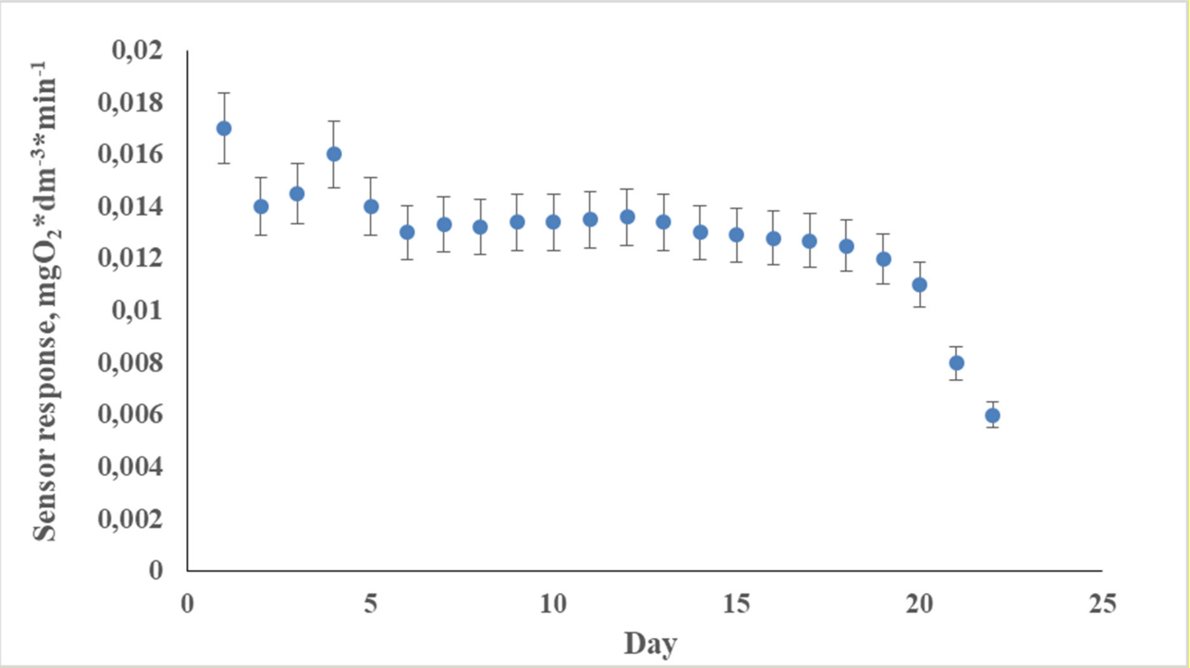

Figure S47. Long-term stability of the formed biosensor

13. Correction factor for enhanced BOD quantification

To account for the consistent underestimation of BOD_5 values by the biosensor (approximately 5.4% on average, as detailed in Section 2.3), a correction factor has been established. This factor is derived from the slope of the regression line in Figure 5 (Main Text):

$$\text{Correction Factor (K)} = 1 / 0.9461 \approx 1.057$$

For practical application and to obtain BOD values aligned with the standard dilution method, the measured biosensor value (BOD (biosensor)) should be multiplied by this factor:

$$\text{BOD (corrected)} = \text{BOD (biosensor)} \times 1.057$$

The use of this correction factor is recommended for applications requiring direct comparability with regulatory standard methods.

14. Quantitative analysis of diffusion limitations

A key parameter for understanding mass transfer in immobilized cell systems is the effectiveness factor (η), which is the ratio of the observed reaction rate with immobilization to the rate without diffusion limitations. The oxygen consumption rate for the immobilized cells in the PHB-xerogel bioreceptor was measured as V(imm) = 0.0092 mg $O_2/dm^3\cdot min$. The oxygen consumption rate for an equivalent concentration of free P. yeei cells in suspension was measured as V(free) = 0.0137 mg $O_2/dm^3\cdot min$.

The effectiveness factor was calculated as follows:

$$\eta = V(imm) / V(free) = 0.0092 / 0.0137 \approx 0.67$$

An η value of 0.67 indicates moderate diffusion limitations. This means the reaction rate in the immobilized state is 67% of what it would be if the cells were completely free from diffusional constraints. This value is considered favorable for biosensor applications, as it confirms that the xerogel matrix provides a biocompatible environment without severely restricting the supply of substrate and oxygen, which is consistent with the biosensor's high performance and fast response time.

15. Example of biosensor operation

The operational principle of the biosensor is based on monitoring the rate of oxygen consumption by immobilized *P. yeei* cells. Figure S48-S51 shows typical response curves recorded after the addition of standard BOD solutions. Upon substrate injection, a rapid decrease in dissolved oxygen concentration is observed, reaching a new steady state within 3-5 minutes.

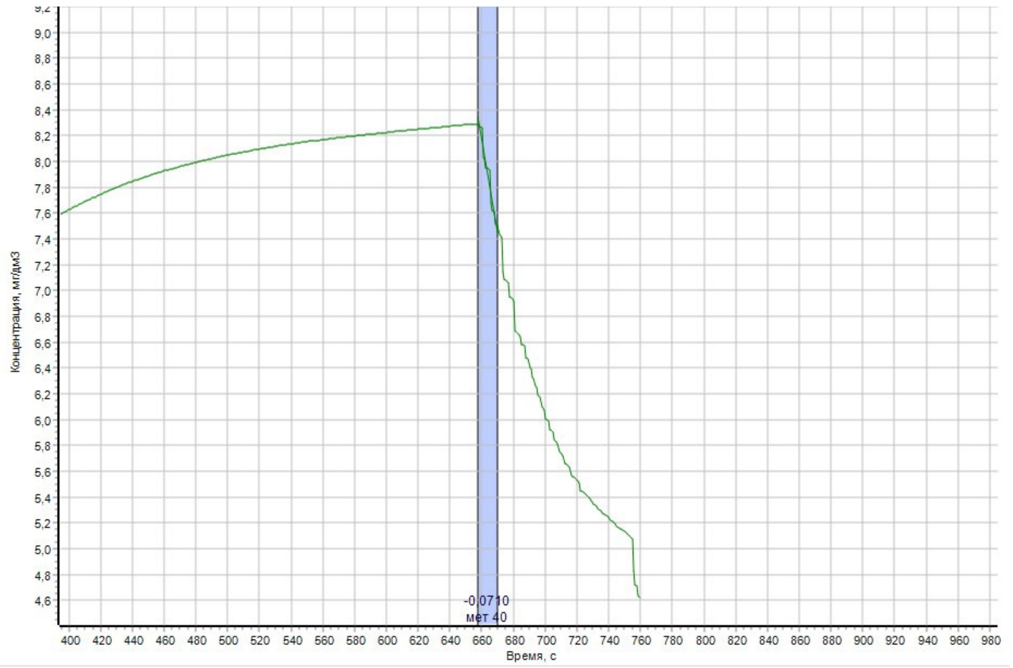

Figure S48. Example of biosensor operation

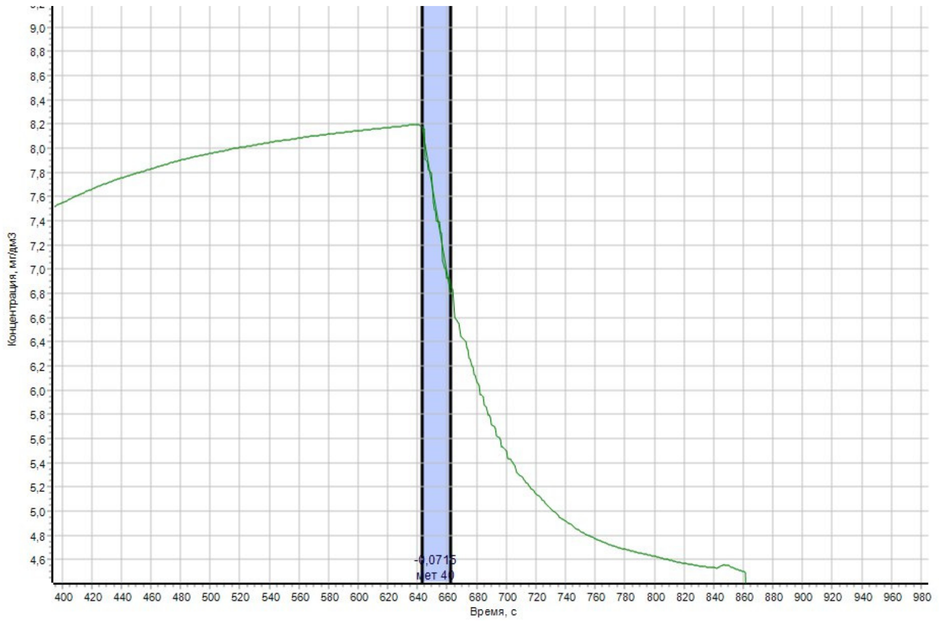

Figure S49. Example of biosensor operation

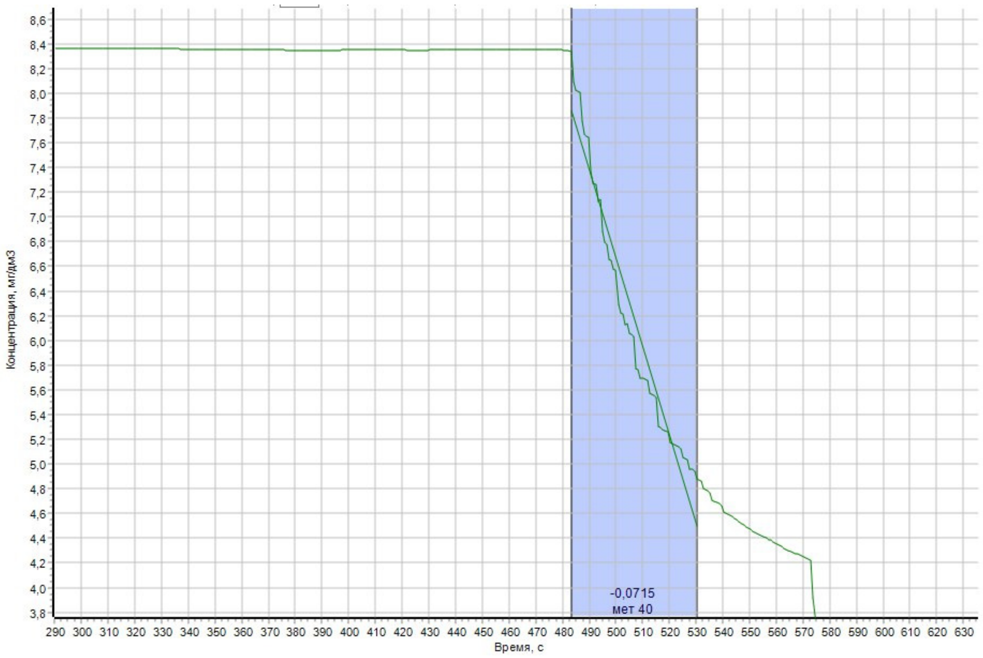

Figure S50. Example of biosensor operation

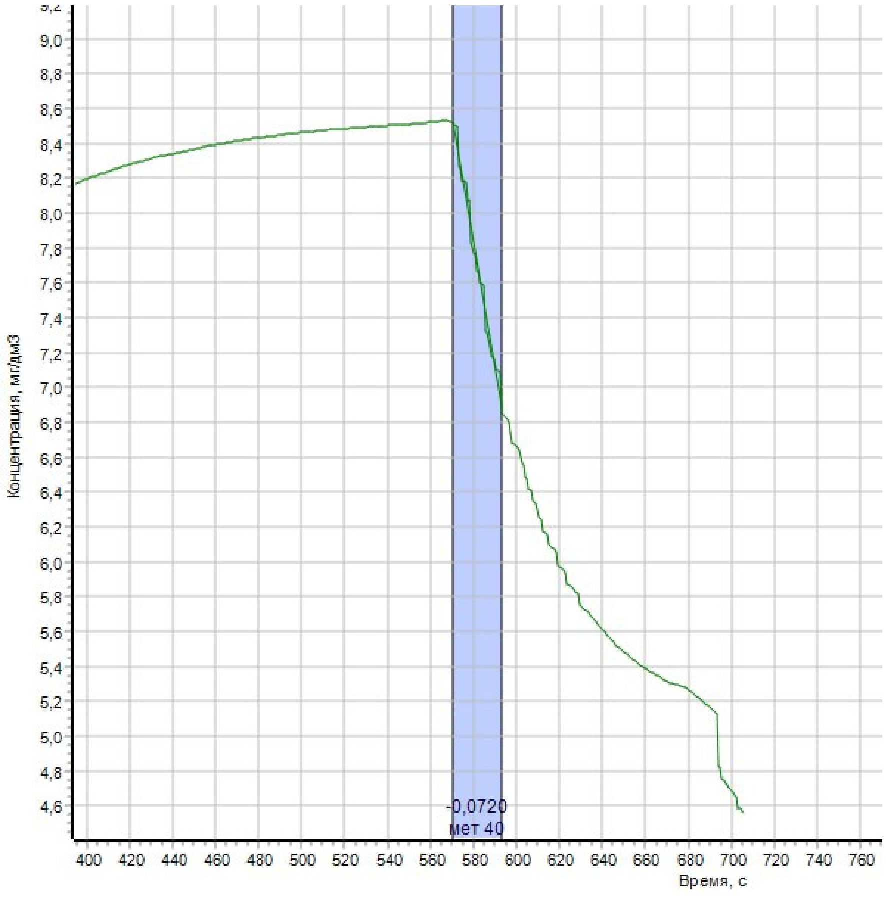

Figure S51. Example of biosensor operation

Reference

1. Kraus, E.; Orf, L.; Starostina, I.; Stoyanov, O. *Improvement of Polymeric Material Adhesion by UV Laser and Plasma Pretreatment*; Shaker Verlag, 2017; ISBN 3-8440-5451-0.
2. Furukawa, T.; Sato, H.; Murakami, R.; Zhang, J.; Noda, I.; Ochiai, S.; Ozaki, Y. Raman Microspectroscopy Study of Structure, Dispersibility, and Crystallinity of Poly(Hydroxybutyrate)/Poly(l-Lactic Acid) Blends. *Polymer* **2006**, *47*, 3132–3140, doi:10.1016/j.polymer.2006.03.010.
3. Acharjee, S.A.; Bharali, P.; Ramachandran, D.; Kanagasabai, V.; Gogoi, M.; Hazarika, S.; Koch, P.J.; Dutta, N.; Maadurshni, G.B.; Manivannan, J.; et al. Polyhydroxybutyrate (PHB)-Based Sustainable Bioplastic Derived from Bacillus Sp. KE4 Isolated from Kitchen Waste Effluent. *Sustainable Chemistry and Pharmacy* **2024**, *39*, 101507, doi:10.1016/j.scp.2024.101507.
4. Hou, J.; Cheng, L.; Zhang, S.; Zhang, X.; Zheng, X.; Zhang, Q. Production of Polyhydroxyalkanoate from New Isolated Bacteria of Acidovorax Diaphorobacter ZCH-15 Using Orange Peel and Its Underlying Metabolic Mechanisms. *Bioresource Technology* **2025**, *418*, 131949, doi:10.1016/j.biortech.2024.131949.
5. Guimarães, T.C.; Araújo, E.S.; Ramos, L.C.; Jesus, A.A.; Leite, S.P.; Bjerk, T.R.; López, J.A.; Hernández-Macedo, M.L. Optimized Bioconversion of Cheese Whey to Poly(Hydroxybutyrate) (PHB) by Mangrove-Isolated Bacillus Cereus. *J Polym Environ* **2025**, *33*, 1881–1896, doi:10.1007/s10924-025-03518-3.
6. ITMO University; Thanh, N.H.; Olekhnovich, R.O.; ITMO University; Uspenskaya, M.V.; ITMO University; Sitnikova, V.E.; ITMO University; Elangwe, C.N.; ITMO University Effect of Polymer Ratio on Thermal Properties of Polyhydroxybutyrate/Polyhydroxyhexanoate. *Bulletin SPbSIT(TU)* **2023**, *66*, 27–30, doi:10.36807/1998-9849-2023-66-92-27-30.
